# Mutual dependency between lncRNA *LETN* and protein NPM1 in controlling the nucleolar structure and functions sustaining cell proliferation

**DOI:** 10.1038/s41422-020-00458-6

**Published:** 2021-01-11

**Authors:** Xianteng Wang, Xiaolin Hu, Wanlu Song, Hui Xu, Zhengtao Xiao, Rongyao Huang, Qingran Bai, Fan Zhang, Yongzhen Chen, Yu Liu, Jianhuo Fang, Xin Li, Qin Shen, Haitao Zhao, Xuerui Yang

**Affiliations:** 1 MOE Key Laboratory of Bioinformatics, Tsinghua University, Beijing, 100084 China; 2 Center for Synthetic & Systems Biology, Tsinghua University, Beijing, 100084 China; 3 School of Life Sciences, Tsinghua University, Beijing, 100084 China; 4 Joint Graduate Program of Peking-Tsinghua-National Institute of Biological Science, Beijing, 100084 China; 5 Brain and Spinal Cord Innovative Research Center of Tongji Hospital, School of Life Sciences and Technology, Tongji University and Frontier Science Research Center for Stem Cells of Ministry of Education, Shanghai, 200092 China; 6 Department of Liver Surgery, Peking Union Medical College Hospital, Chinese Academy of Medical Sciences & Peking Union Medical College (CAMS & PUMC), Beijing, 100730 China

**Keywords:** Long non-coding RNAs, Cell division

## Abstract

Fundamental processes such as ribosomal RNA synthesis and chromatin remodeling take place in the nucleolus, which is hyperactive in fast-proliferating cells. The sophisticated regulatory mechanism underlying the dynamic nucleolar structure and functions is yet to be fully explored. The present study uncovers the mutual functional dependency between a previously uncharacterized human long non-coding RNA, which we renamed *LETN*, and a key nucleolar protein, NPM1. Specifically, being upregulated in multiple types of cancer, *LETN* resides in the nucleolus via direct binding with NPM1. *LETN* plays a critical role in facilitating the formation of NPM1 pentamers, which are essential building blocks of the nucleolar granular component and control the nucleolar functions. Repression of *LETN* or NPM1 led to similar and profound changes of the nucleolar morphology and arrest of the nucleolar functions, which led to proliferation inhibition of human cancer cells and neural progenitor cells. Interestingly, this inter-dependency between *LETN* and NPM1 is associated with the evolutionarily new variations of NPM1 and the coincidental emergence of *LETN* in higher primates. We propose that this human-specific protein–lncRNA axis renders an additional yet critical layer of regulation with high physiological relevance in both cancerous and normal developmental processes that require hyperactive nucleoli.

## Introduction

Comprehensive annotations of transcriptomes have revealed a vast number of long non-coding RNAs (lncRNAs) in various species.^1–5^ For the past years, extensive studies have uncovered hundreds of lncRNAs in human cells with various molecular and cellular functions.^6,7^ Specifically, many of these lncRNAs have been emerging as fine-tuners of key pathways related to a variety of processes, such as development^5^ and complex diseases such as cancer.^8–10^ Among them, physiologically relevant lncRNAs with fundamental and indispensable functions are still rare.

The nucleolus is a non-membrane-bound subnuclear compartment, which is a crucial organelle for fundamental processes such as rDNA transcription and ribosome biogenesis.^11^ Dysregulation of the nucleolar organization and functions thereby results in a multitude of cellular and physiological disorders, such as ribosomopathies, aging, and cancer.^12,13^ The major function of the nucleolus, rDNA transcription by polymerase I (Pol I), is subjected to complicated regulation, which involves several recently discovered lncRNAs in human cells, including PAPAS,^14,15^ SLERT,^16^ and HOXB-AS3.^17^ These lncRNAs function mainly on the Pol I-mediated rDNA transcription machinery rather than on the nucleolus itself. In the present study, we identified a lncRNA (transcript ID: ENST00000564237.1, gene name: RP11-196G18.22) that directly controls the structural organization and major functions of the nucleolus via a key nucleolar protein NPM1 (also named B23 or nucleophosmin).

Known as an essential and the most abundant protein in the nucleolus,^18–20^ NPM1 is localized in the granular component (GC) and controls the structured formation of the nucleolus.^20–23^ NPM1 is a well-characterized multifunctional protein that plays key roles in rDNA transcription in the nucleolus,^19,20,24^ nucleosome assembly as a histone chaperone protein,^25,26^ and ribosome biogenesis by shuffling ribosomal proteins.^27,28^ Due to its critical position in multiple biological processes, NPM1 has been shown to be essential for embryogenesis^29^ and tumorigenesis.^30,31^

The expression of lncRNA RP11-196G18.22 is relatively low in most human adult normal tissues but elevated in multiple types of tumors and in embryonic tissues^32^ (Supplementary information, Fig. S1a). The molecular function of the lncRNA RP11-196G18.22 has not yet been studied. Here, we show that the major functions of NPM1, such as promoting rRNA synthesis and chromatin condensation, all depend on the lncRNA RP11-196G18.22. Specifically, by directly binding with NPM1, the lncRNA is concentrated in the nucleolus and plays a critical role for the formation of NPM1 oligomers, which are main building blocks of the GC of the nucleolus and required for the functional activities of NPM1.^20,22,33–37^ Indeed, repression of the lncRNA RP11-196G18.22 generated phenotypes identical to those observed with NPM1 knockdown, i.e., disordered morphology and functions of the nucleolus and strong inhibition of proliferation of human cancer cells and neuronal progenitor cells (NPCs). We therefore propose to rename the lncRNA RP11-196G18.22 as *LETN* (lncRNA essential for tumor cell proliferation via NPM1).

In summary, we have uncovered the mutual functional dependency between the key multi-functional protein NPM1 and a previously uncharacterized lncRNA. Interestingly, our comparative genomic analyses further showed that this inter-dependency between *LETN* and NPM1 is associated with the evolutionarily new variations of NPM1, which are coincidental with the emergence of *LETN* in higher primates. Therefore, this human-specific *LETN*-NPM1 axis possessed a highly critical position in the fundamental processes in the nucleolus by implementing an additional yet critical layer of regulation. This is rare to see and renders the high physiological relevance of this lncRNA in cancer and potentially neuronal development.

## Results

### lncRNA *LETN* is critical for cancer cell proliferation and tumor development

**Fig. 1:**
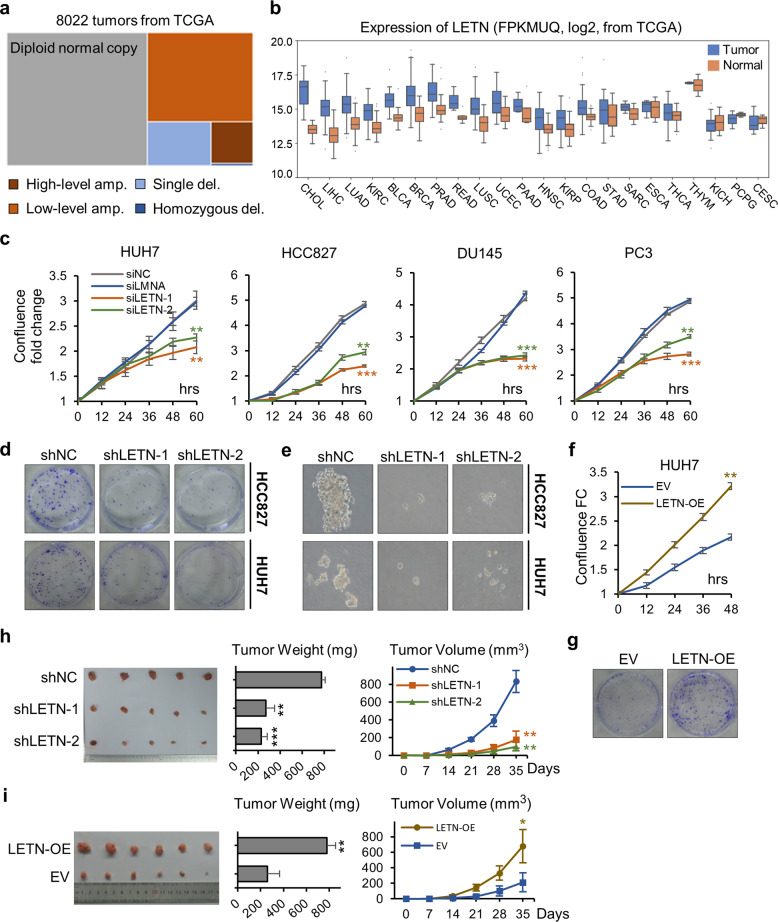
Expression of *LETN* in cancers and its function in tumor cell proliferation. **a** Copy number variations of the *LETN* gene locus in pan-cancer tumors. The data were obtained from 22 cancer types in TCGA. Refer to Supplementary information, Fig. S1b for the data of each specific cancer type. **b** Box plots showing the RNA expression levels of *LETN* (FPKM after upper-quantile normalization) in tumors of different cancer types and in the corresponding normal tissues. The data were obtained from TCGA. The 22 cancer types were sorted from left to right according to the significance of the *LETN* differential expression between the tumors and the paired normal tissues. **c** Cell proliferation curves of HUH7, HCC827, DU145 and PC3 cells upon siRNA-mediated knockdown of *LETN*. The *y-*axis, cell confluence fold-change (FC) in relative to the confluence at time 0. The error bars represent the ± SD of three biological replicates. **d**, **e** Anchorage-dependent (**d**) and -independent (**e**) colony formation of HCC827 and HUH7 cells upon shRNA-mediated knockdown of *LETN*. **f** Cell proliferation curves of HUH7 and HCC827 cells with stable overexpression of *LETN*. The error bars represent the ± SD of three biological replicates. **g** Anchorage-dependent colony formation of HUH7 and HCC827 cells with stable overexpression of *LETN*. **h**, **i** Three million of the HUH7 cells with lentivirus-mediated stable *LETN* knockdown (**h**) or 1 million of the cells with stable overexpression of *LETN* (**i**) were implanted into the nude mice (*n* = 5 for knockdown and *n* = 6 for overexpression) for xenograft tumor models. Images, weights, and growth records of the tumors are being presented. The error bars represent the ± SD of the tumors.

The expression of lncRNA *LETN* (previously named as RP11-196G18.22, at chr1:149,844,498–149,849,024 of GRCh38/hg38) is relatively high in the human embryonic tissues but then largely repressed in most adult normal tissues^32^ (Supplementary information, Fig. S1a). The gene locus of *LETN* exhibits frequent copy number amplifications in liver hepatocellular carcinoma (LIHC) and many other types of cancer (Fig. 1a; Supplementary information, Fig. S1b). Consistently, RNA expression of *LETN* was significantly upregulated in tumors compared to their adjacent normal tissues in a broad range of cancers, including bile duct, liver, lung, and kidney (Fig. 1b).

In cancer cell lines such as HUH7 (liver cancer) and HCC827 (lung cancer), *LETN* has fairly high RNA copy numbers among lncRNAs (nearly 200 copies per cell, as shown by quantitative PCR with standard RNA spike-in as the reference) (Supplementary information, Fig. S2). Although the genome annotation of *LETN* has been available, we performed 5′ and 3′ rapid amplification of cDNA ends (RACE) to precisely identify the two ends of *LETN* in HUH7 cells. The 3′ RACE confirmed the previously annotated 3′ end of *LETN* (chr1:149,844,498), but the amplified sequence from 5′ RACE was 23-nt shorter than the reference at the 5′ end (Supplementary information, Fig. S3a). Therefore, the genome annotation of *LETN* may be revised to chr1:149,844,498–149,849,001. However, as many of the transcription start sites are known to vary in different cell contexts,^38,39^ updating the genome annotation of *LETN* is not urgently needed. Finally, the lengths of the amplified products from 5′ and 3′ RACE have supported the annotated full length (~4.5 knt) of *LETN* (Supplementary information, Fig. S3a), which was further confirmed by northern blots of *LETN* in control and *LETN* knockdown HUH7 cells (Supplementary information, Fig. S3b).

siRNA-mediated knockdown of *LETN* (Supplementary information, Fig. S4a) significantly repressed cell proliferation rates in various cancer cell lines, including HUH7, HCC827, PC3 and DU145 (prostate cancer) (Fig. 1c). Lentivirus-mediated long-term knockdown of *LETN* with shRNA (Supplementary information, Fig. S4b) reduced anchorage-dependent or -independent colony formation from the cells (Fig. 1d, e). Gene knockout of *LETN* via CRISPR–Cas9 in HUH7 cells (80% reduction of *LETN* expression after enrichment of the cells with positive Cas9–sgRNA vector transfection, Supplementary information, Fig. S5a) also significantly reduced cell proliferation and colony formation (Supplementary information, Fig. S5b, c). Additionally, the reduction of cell proliferation rate was also confirmed by *LETN* knockdown mediated by antisense oligonucleotides (ASO) (Supplementary information, Fig. S6a, b). On the other hand, lentivirus-mediated stable overexpression of *LETN* (approximately tenfold increase) in HUH7 cells resulted in significant increases in cell proliferation and colony formation (Fig. 1f, g).

Finally, in multiple xenograft tumor models established with cancer cell lines including HUH7 and HCC827, stable knockdown of *LETN* greatly inhibited tumor growth (Fig. 1h; Supplementary information, Fig. S7), whereas its overexpression in HUH7 granted significant growth advantages to the tumors (Fig. 1i). From the results above, it is clear that the lncRNA *LETN* plays a potent tumor-promoting role in multiple cancer cells, and even partial repression of *LETN* could lead to significant arrest of cell proliferation and tumor growth.

### lncRNA *LETN* binds to and coaggregates with NPM1 in the nucleolus

**Fig. 2:**
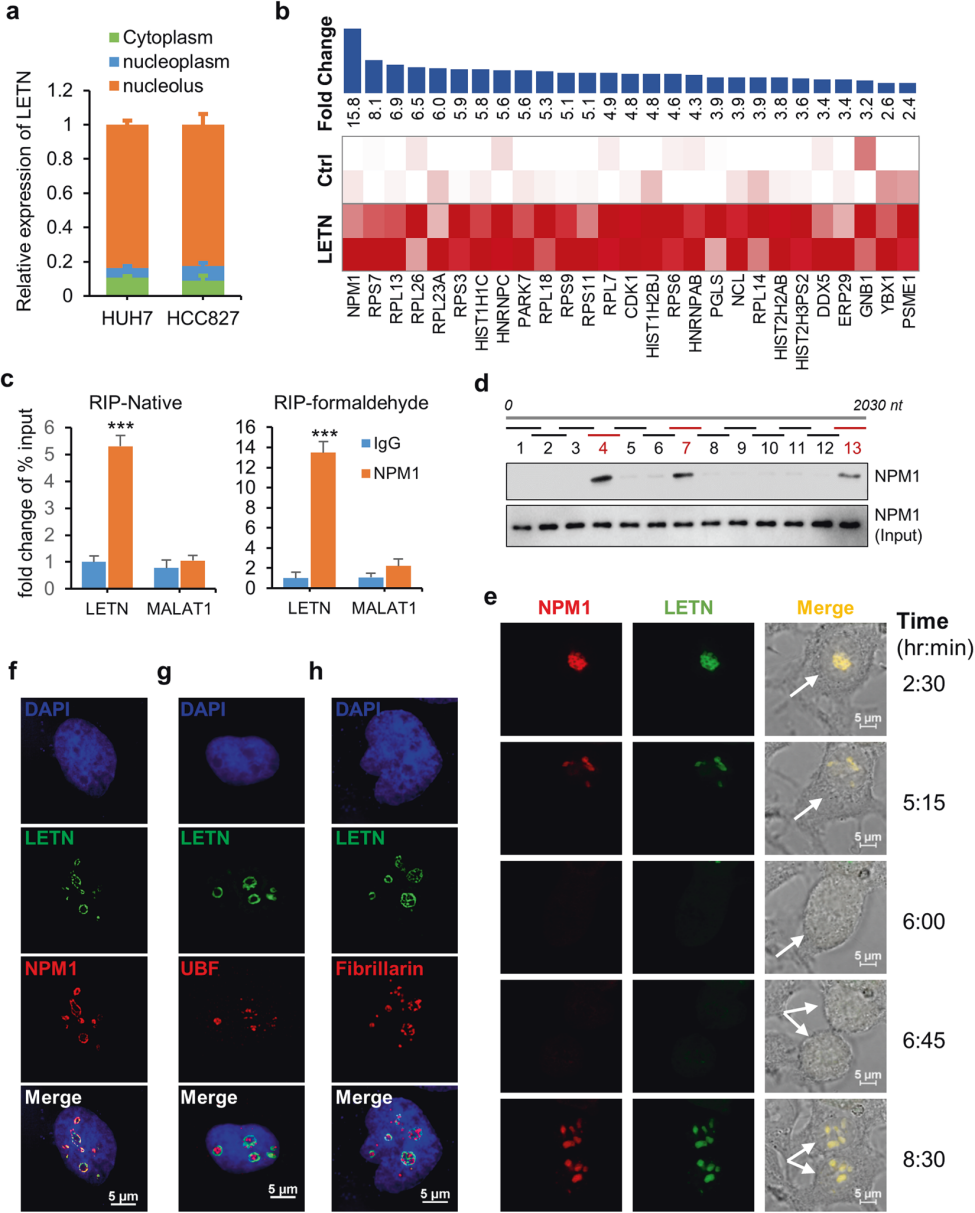
Direct interaction between *LETN* and NPM1. **a** RT-qPCR of *LETN* in the cytoplasmic, nucleoplasmic and nucleolar fractions of HUH7 and HCC827 cells. Shown in Supplementary information, Fig. S9, GAPDH, U6 and pre-rRNA were used as markers of the three compartments, respectively. **b** Mass spectrometry profiles of the proteins pulled down by MS2-tagged *LETN* RIP in HUH7 cells, compared to the MS2-RIP negative control. For two biological replicates, all the proteins detected by mass spectrometry analysis in either *LETN*-MS2-RIP (*LETN*) or MS2-RIP (ctrl) are included in the table. **c** qPCR of *LETN* and *MALAT1* after native and formaldehyde cross-linking NPM1-RIP in HUH7 cells. *MALAT1* was used as a negative control. The error bars represent the ± SD of three replicates. **d** Interactions between fragmented *LETN* and NPM1. Thirteen biotin-labeled RNA fragments (about 180 nt each) covering the first 2030 nt of *LETN* were used for protein pull-down in HUH7 cells, followed by western blotting of NPM1. See Supplementary information, Fig. S12a, b for more data. **e** Representative images from time-lapse microscope of a dividing HUH7 cell, showing the mCherry-labeled NPM1 (red) and MS2-tagged *LETN* marked by MS2-GFP fusion protein (green). Twenty-four hours after transfections of plv-NPM1-mcherry, pMS2-GFP, and pcDNA3.1-*LETN*-MS2, the cells were put under a microscope and photographed every 15 min for up to 12.5 h. Refer to Supplementary information, Fig. S13b and Video S1 for the full data. SIM images showing the nucleus staining by DAPI (blue), IF (red) of NPM1 (**f**), UBF (**g**), or fibrillarin (**h**), and MS2-tagged *LETN* marked by MS2-GFP (green) in the HUH7 cells after *LETN* knockout and re-expression with the pcDNA3.1-*LETN*-MS2 plasmid. Quantifications of the colocalizations between *LETN* and the three proteins are provided in Supplementary information, Fig. S13c.

RNA Fluorescence in situ hybridization (FISH) and qPCR of the cytoplasmic and nuclear fractions showed that the endogenous *LETN* is predominantly, if not only, localized in the cell nucleus (Supplementary information, Fig. S8a, b). Note that the siRNA transfection resulted in significant knockdown of *LETN* in the nucleus, as confirmed by both FISH and qPCR (Supplementary information, Fig. S8a, b). In addition, the distribution of *LETN* in the nucleus and the DAPI staining strongly indicated that *LETN* is enriched in the nucleolus (Supplementary information, Fig. S8a). This was directly confirmed by qPCR assays of *LETN* in the three cell fractions (cytoplasm, nucleoplasm, and nucleolus) (Fig. 2a), of which the purities were validated by qPCR assays of GAPDH, U6, and pre-rRNA, respectively (Supplementary information, Fig. S9).

Next, we used the technique of MS2 tagging followed by mass spectrometry^40^ to explore the proteins that bind to *LETN* (schematic description of the assay provided in Supplementary information, Fig. S10a). The results showed that NPM1 was the most enriched binding partner of *LETN* (Fig. 2b). It is worth noting that more than half of the other proteins pulled out by the MS2-tagged *LETN* (85 out of 152) were also found in the proteins pulled down by NPM1 immunoprecipitation (176 in total) (Supplementary information, Fig. S10b), which is a strongly significant overlap (*P*-value close to 0).

On the other hand, the NPM1-RNA immunoprecipitation (RIP) assay, i.e., immunoprecipitation of the endogenous NPM1 followed by qPCR, confirmed the binding of NPM1 to *LETN* under both native and formaldehyde cross-linking conditions (Fig. 2c). Another series of RIP assays with multiple exogenously expressed truncated forms of NPM1 showed that the C-terminal domain of NPM1 is essential for directly binding with *LETN* (Supplementary information, Fig. S11a). NPM1 is one of the most abundant proteins in the nucleolus, and it is frequently used as a nucleolar marker.^41–43^ The nucleolar localization signal (NoLS) of NPM1 is located in the C-terminal region of NPM1.^44^ Therefore, as expected, the two fragments NPM1.1 and NPM1.2 failed to concentrate in the nucleolus (Supplementary information, Fig. S11b), whereas the fragment NPM1.3 and the full-length NPM1 were highly enriched in the nucleoli (Supplementary information, Fig. S11b). To exclude the possibility of NPM1–*LETN* interaction simply due to their colocalization in the nucleoli, we performed an in vitro binding assay with biotin-labeled *LETN* and the fragmented and full-length NPM1 proteins. As shown in Supplementary information, Fig. S11c, the two NPM1 fragments (NPM1.1 and NPM1.2) indeed have lost the binding capacity with *LETN*, whereas the C-terminal fragment (NPM1.3) and the full-length NPM1 showed clear binding with *LETN*.

Next, to identify the NPM1-binding region in *LETN*, we used a series of biotin-labeled fragments of the lncRNA for protein pull-down, followed by western blotting for NPM1. The first round of fragmentation narrowed down the binding to the region of 500–2000 nt of *LETN* (Supplementary information, Fig. S12a). The second round, designed for the region of 1–2000 nt and with a higher resolution, further identified three NPM1 binding domains, i.e., 500–680 nt (fragment 4), 950–1130 nt (fragment 7), and 1850–2030 nt (fragment 13) (Fig. 2d). Removing any of these three domains indeed slightly attenuated NPM1 binding, but only the simultaneous deletion of all three domains could completely block NPM1 binding (Supplementary information, Fig. S12b).

We were not able to detect any significantly conserved motifs among the three *LETN* fragments identified above (4, 7, and 13). We suspect that like many other lncRNAs, it is the higher order structure of the RNA that has enabled its interaction with NPM1. In addition, although the *LETN* fragments 4, 7, and 13 were responsible for the direct interaction with NPM1, simply expressing these three fragments together has no rescuing effect on cell proliferation after knockdown of the endogenous *LETN* (Supplementary information, Fig. S12c). In fact, the truncated *LETN* of the first 1000 nt also showed no rescuing effect. By contrast, the first 2000 or 3000 nt of *LETN* significantly rescued cell proliferation, but still not as potent as the *LETN* full length. Based on these observations, it is a plausible hypothesis that the full molecular and cellular functions of *LETN* depend on its high-order structure, instead of some short sequence motifs.

Finally, immunofluorescence staining of endogenous NPM1 further showed its clear coaggregation and colocalization with *LETN* in the nucleoli (Supplementary information, Fig. S13a). In fact, taking one field of the time-lapse microscopy as an example, the exogenously expressed NPM1 and *LETN* exhibited perfectly synchronized and colocalized dissociation and aggregation in the nucleus before and after cell division (Fig. 2e; Supplementary information, Fig. S13b and Video S1). More examples of the time-lapse microscopy images are provided in Supplementary information, Videos S2–5. NPM1 is predominantly localized in the GC compartment of the nucleolus.^11,22,45^ High-resolution IF of the endogenous NPM1 and RNA FISH of *LETN* with structured illumination microscopy (SIM) illustrated colocalization of *LETN* and NPM1 mostly in the peripheral regions of the nucleolus (Fig. 2f), apparently the GC. This strong colocalization was nicely supported by the Pearson’s correlation coefficients (PCC)^46^ between the distributions of the two factors in multiple cells (Supplementary information, Fig. S13c). On the other hand, UBF as a marker of the nucleolus fibrillar center (FC)^45,47^ and fibrillarin as a marker of the dense fibrillar component (DFC)^11,48^ were found in sparse spots inside of the nucleoli, which are almost completely excluded from the locations of *LETN* in the GC (Fig. 2g, h). This was supported by the low PCC values between *LETN* and either of the two proteins (Supplementary information, Fig. S13c), which were significantly lower than those between *LETN* and NPM1.

In summary, both the in vitro and in vivo assays above have demonstrated the direct binding between NPM1 and *LETN*, which nicely congregate in the GC of cell nucleoli. This inspired us to further study whether the potential molecular functions of the lncRNA *LETN* are related to the protein NPM1 (and vice versa).

### Repression of the lncRNA *LETN* or the protein NPM1 results in identical phenotypes

As a nucleolar phosphoprotein, NPM1 is well known for playing crucial roles in a multitude of important intracellular processes, such as rDNA transcription, ribosome maturation, and nucleosome assembly.^49^ As a result, NPM1 is essential for embryonic development and tumorigenesis.^29^ Indeed, knockdown of NPM1 significantly reduced the proliferation rates of HUH7 and HCC827 cells (Supplementary information, Fig. S14), which was similar to the inhibitory effect of *LETN* repression on cell proliferation (Fig. 1).

**Fig. 3:**
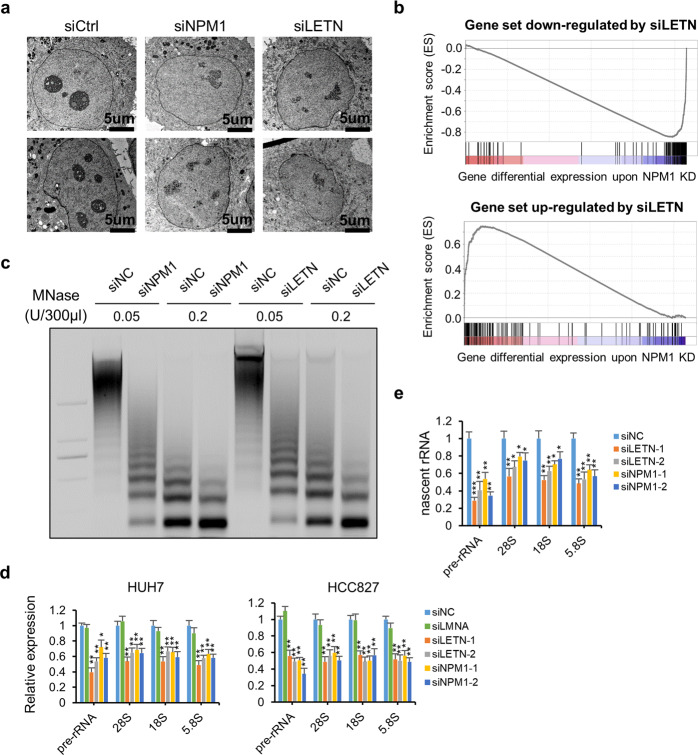
Repression of the lncRNA *LETN* or the protein NPM1 leads to the same phenotypes. **a** Images of the nucleoli (obtained with TEM at 80 kV, 7000×) of HUH7 cells under the control and *LETN* or NPM1 knockdown conditions. **b** Gene set enrichment analysis plots showing enrichments of the gene sets that was downregulated or upregulated upon *LETN* knockdown in HUH7 cells, on the background of gene differential expression profile upon NPM1 knockdown. **c** MNase digestion assay. The chromatin of HUH7 cells was partially digested with MNase at different concentrations. The resulted DNA fragments were separated and visualized by gel electrophoresis. **d** Relative expression levels of pre- and mature rRNAs, normalized to beta-actin, measured by RT-qPCR in HUH7 and HCC827 cells upon *LETN* or NPM1 knockdown. Random siRNA or siLMNA were used as negative controls. Data show means ± SD of three biological replicates. **e** Relative expression levels of the newly synthesized nascent pre- and mature rRNAs, which were marked by EU and measured by RT-qPCR, in HUH7 cells upon *LETN* or NPM1 knockdown. Newly synthesized beta-actin was used as a house-keeping gene for normalization. Data show means ± SD of three biological replicates.

Known as an essential and the most abundant protein in the nucleolus,^19,20,24^ NPM1 is critical for the structured formation of the nucleolus.^20–23^ As shown by electron microscopy images, knockdown of NPM1 resulted in largely disturbed nucleolar structures (Fig. 3a, more examples in Supplementary information, Fig. S15), which is consistent to the literature.^23,50^ Strikingly, similarly distorted nucleolus morphology was also observed upon knockdown of the lncRNA *LETN* (Fig. 3a, more examples in Supplementary information, Fig. S15).

In addition to the consistent phenotypic responses at the cellular level, we then tested whether repression of *LETN* or NPM1 may have affected the same molecular processes. From the global perspective, knockdown of *LETN* and NPM1 resulted in similar shifts of the gene expression profiles (Fig. 3b; Supplementary information, Fig. S16a), which were translated to the similar biological functions being perturbed by knockdown of *LETN* or NPM1 (Supplementary information, Fig. S16b). Specifically, knockdown of the lncRNA or the protein led to repression of the genes with highly focused functions such as chromatin and nucleosome assembly, DNA packaging, and protein–DNA complex assembly (Supplementary information, Fig. S16b).

NPM1 has been well recognized as a histone chaperone protein in the nucleolus,^23,51–53^ and a major function of NPM1 is facilitating nucleosome assembly and chromatin condensation.^27^ Knockdown of NPM1 dramatically increased the sensitivity of the chromatin to micrococcal nuclease (MNase) digestion, which indicates a substantial decrease in chromatin compaction (Fig. 3c). Consistently, knockdown of the lncRNA *LETN* resulted in a similar reduction in chromatin condensation (Fig. 3c).

Another major function of NPM1 is driving rDNA transcription and ribosome maturation.^19,20,24^ siRNA- or ASO-mediated knockdown or CRISPR-mediated knockout of *LETN* resulted in decreased expression of 47S pre-rRNA and mature rRNA (Fig. 3d; Supplementary information, Fig. S17a, b). This is consistent with the effect of NPM1 knockdown or knockout (Fig. 3d; Supplementary information, Fig. S17a). Northern Blots confirmed that the pre-rRNA was downregulated by knockdown of *LETN* or NPM1 (Supplementary information, Fig. S17c). Next, we showed that the repression of *LETN* or NPM1 reduced the newly synthesized nascent pre- and mature rRNAs, which were marked by the uridine analog 5-ethynyluridine (EU) (Fig. 3e). Overexpression of the lncRNA *LETN* led to elevated levels of the total pre- and mature rRNAs (Supplementary information, Fig. S17d) and the newly synthesized nascent pre- and mature rRNAs (Supplementary information, Fig. S17e). Finally, a dual-luciferase reporter system for the rDNA promoter showed that knockdown of *LETN* or NPM1 resulted in similarly reduced rDNA promoter activities (Supplementary information, Fig. S17f). It is worth nothing that when the general transcription was inhibited by Actinomycin D, the degradation rates of the rRNAs were unaffected by knockdown of either *LETN* or NPM1 (Supplementary information, Fig. S18). Taken together, the results above have illustrated the strong promotive effect of *LETN* in the process of rRNA synthesis, which is comparative to that of NPM1.

Inspired by the results above, and given the direct binding between *LETN* and NPM1, we proposed a hypothesis that the well-recognized physiological functions of NPM1 may depend on the lncRNA *LETN*.

### Oligomerization and nucleolar organization of NPM1 depend on *LETN*

**Fig. 4:**
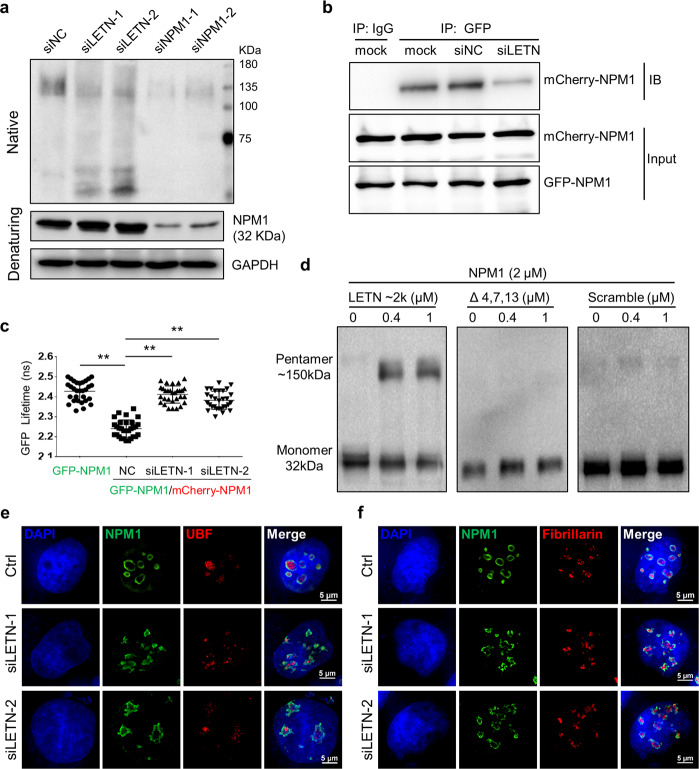
The oligomerization and structural organization of NPM1 is dependent on *LETN*. **a** Upper, image of native PAGE with anti-NPM1 showing oligomeric and monomeric NPM1 proteins in HUH7 cells under conditions of *LETN* or NPM1 knockdown. Bottom, regular denaturing western blots showing the total protein level of NPM1. **b** GFP- and mCherry-tagged NPM1 were introduced into HUH7 cells by transfection. Co-immunoprecipitation of the exogenously expressed GFP- and mCherry-tagged NPM1 was performed to show the interactions between NPM1 proteins in control and *LETN* knockdown cells. **c** GFP- and mCherry-tagged NPM1 were introduced into HUH7 cells by transfection. The lifetime of GFP was quantified for 30 randomly selected cells, which indicates efficiency of energy transfer from GFP to mCherry. Cells with only the GFP-NPM1 served as a reference, in which there is no energy transfer as the acceptor fluorophore (mCherry) is absent. **d** In vitro assays with purified NPM1 monomeric protein, which was incubated for about 40 min with the first ~2000 nt of *LETN*, the same *LETN* sequence but with the 3′ NPM1-binding fragments removed, or scrambled RNA with a similar length. Images of native PAGE with anti-NPM1 show the oligomeric and monomeric NPM1 proteins. SIM images showing the nucleus staining by DAPI (blue) and IF of NPM1 (green), UBF (red, **e**), and fibrillarin (red, **f**) in HUH7 cells.

NPM1 has a conserved N-terminal oligomerization domain that mediates pentamerization of the protein. Multiple studies have shown that oligomerization of NPM1 is critical for its biological functions^44,52,54–58^ and that the disruption of the oligomeric status resulted in loss of its functions, such as histone binding and nucleosome assembly,^33,34^ rDNA transcription and ribosome maturation.^35–37,59^ Therefore, we tested the oligomerization of NPM1 with native polyacrylamide gel electrophoresis (PAGE), in which proteins retain their folded conformations and thereby oligomers can be separated from monomers. In control cells, endogenous NPM1 presents predominantly as polymers (likely pentamers according to the protein markers, Fig. 4a). Strikingly, upon knockdown of *LETN*, the NPM1 oligomers (> 135 KDa) were greatly disrupted, and the monomeric NPM1 (~30 KDa) became the dominant form (Fig. 4a), indicating that the in vivo oligomerization of NPM1 requires the lncRNA *LETN*. Note that the total protein abundance of NPM1 was unaffected by *LETN*, as shown by the regular western blots from a denaturing procedure (Fig. 4a)

Co-immunoprecipitation of exogenously expressed GFP- and mCherry-tagged NPM1 also confirmed the interaction between different NPM1 molecules, which was largely disrupted by knockdown of *LETN* (Fig. 4b). To further directly assess the closeness between NPM1 proteins, we used fluorescence lifetime imaging microscopy in combination with Förster resonance energy transfer (FLIM-FRET) assay. FLIM-FRET quantifies the shortening of fluorescence donor lifetime as a measurement of FRET efficiency, which indicates the proximity of the donor and acceptor. As shown in Fig. 4c, exogenous expression of mCherry-tagged NPM1 significantly reduced the GFP lifetime in the cells expressing GFP-tagged NPM1, which confirms the closeness between NPM1 proteins under normal conditions. However, knockdown of *LETN* completely restored the GFP lifetime back to the level of the control, in which the mCherry-NPM1 as the energy acceptor is absent (Fig. 4c). This result again indicates that the close interaction between NPM1 proteins requires their binding partner, the lncRNA *LETN*.

In addition to the in vivo experiments above, in vitro assays also showed that the *LETN* RNA fragment of just the first 2000 nt, synthesized by in vitro transcription, greatly promoted pentamerization of the purified monomeric NPM1 protein in vitro, whereas the scrambled RNA with similar length failed to do so (Fig. 4d). Importantly, *LETN* sequence lacking the three NPM1-binding fragments (4, 7, and 13 as indicated in Fig. 2d) also failed to facilitate the NPM1 pentamerization (Fig. 4d).

NPM1 is predominantly localized in the GC of the nucleolus.^11,22,45,60,61^ Furthermore, it is the NPM1 pentamer that serves as the main building block of the GC.^20–22^ Indeed, high-resolution IF of the endogenous NPM1 with SIM illustrated roughly globular distribution patterns of NPM1 in the nucleoli of the control cells (Fig. 4e, f; 3D structures of the nucleoli reconstructed with SIM images of NPM1 are shown in Supplementary information, Video S6). However, upon knockdown of *LETN*, such highly organized distributions of NPM1, which mark the nucleolus GC morphology, were remarkably disrupted (Fig. 4e, f; 3D structures of the nucleoli are shown in Supplementary information, Video S8). Such disorder of the GC morphology by *LETN* knockdown was confirmed by the distribution of nucleolin (NCL), which is another marker of the GC (Supplementary information, Fig. S19; 3D structures of the nucleoli shown in Supplementary information, Videos S7 and S9). These data are also nicely consistent with our previous observation in Fig. 3a that knockdown of *LETN* or NPM1 resulted in distorted nucleolar morphology. In addition, we noted that knockdown of *LETN* did not seem to disturb the FC and DFC of the nucleolus, which were marked by UBF and Fibrillarin, respectively (Fig. 4e, f; Supplementary information, Fig. S19 and Videos S6–S9).

Taken together, the results above showed that oligomerization of NPM1 in cells requires the lncRNA *LETN*. Under the condition of *LETN* repression, the NPM1 proteins can still be concentrated in the nucleoli, which is expected given the NoLS in the C-terminal region of NPM1.^44^ However, as the polymerized NPM1, instead of the monomer, is critical for the formation of nucleolus, the nucleolus, specifically the GC, could not maintain its organized structure upon repression of *LETN*.

### Mutual dependency between *LETN* and NPM1 in their molecular functions

**Fig. 5:**
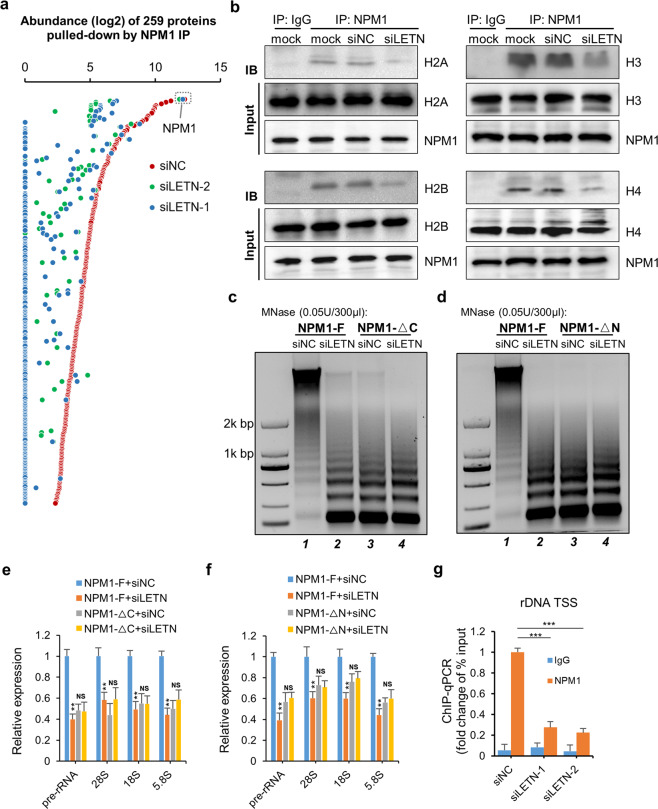
Repression of *LETN* diminished the major molecular functions of NPM1. **a** Immunoprecipitation of NPM1 followed by mass spectrometry analysis of the proteins pulled down, in control and *LETN* knockdown HUH7 cells. As expected, the most abundant protein, NPM1, was consistent in control and *LETN* knockdown cells, thereby serving as a positive control. **b** Immunoprecipitation of NPM1 followed by immunoblotting of several histone proteins in HUH7 cells, with or without *LETN* knockdown. NPM1^–/–^ HUH7 cells were established by CRISPR-mediated gene knockout. Full-length NPM1 (NPM1-F), C-terminus (NPM1-ΔC, 1–189 aa remaining, panel **c**, **e**) or N-terminus (NPM1-ΔN, 122–294 aa remaining, panel **d**, **f**) truncated NPM1 was then reintroduced into the NPM1^–/–^ cells. The chromatin of these cells upon siRNA-mediated knockdown of *LETN* was partially digested with MNase (0.05 U/300 µL). The resulted DNA fragments were separated and visualized by gel electrophoresis (**c**, **d**). Relative expression levels of pre- and mature rRNAs were measured by RT-qPCR upon *LETN* knockdown and normalized to the housekeeping gene beta-actin (**e**, **f**). The statistical significance levels were obtained by pair-wise comparisons between siNC and si*LETN* (**e**, **f**). Data show means ± SD of three biological replicates. **g** ChIP-qPCR assay of the rDNA promoter sequence after immunoprecipitation with anti-NPM1 in HUH7 cells. Data show means ± SD of three biological replicates.

As the major molecular functions of NPM1 were carried out by the oligomerized protein, which is indeed the dominating conformation of NPM1 (Fig. 4), it is a plausible hypothesis that the multiple molecular functions of NPM1 depend on the lncRNA *LETN*. Proteomic profiling of the NPM1-binding proteins showed that knockdown of the lncRNA *LETN* reduced the binding affinity of NPM1 to almost all of its binding partners (Fig. 5a), which are mostly histone proteins, ribosomal proteins, and some other proteins involved in RNA processing. (Supplementary information, Fig. S20). As a validation, our co-immunoprecipitation results clearly showed that the interactions between NPM1 and the histone proteins H2A, H2B, H3, and H4 were all largely weakened upon knockdown of *LETN* (Fig. 5b).

In fact, as introduced earlier in Fig. 3, one of the major functions of NPM1, facilitating nucleosome assembly and chromatin condensation, depends on its activity as a histone chaperone protein. It has been reported that both the N- and C-terminal domains of NPM1 are essential for its oligomerization and functional activation.^62^ Therefore, as expected, after gene knockout of *NPM1* with CRISPR, the re-expression of the N- or C-terminally truncated NPM1 (NPM1-ΔN/C, Supplementary information, Fig. S21), instead of the full-length protein, failed to restore the compactness of the chromatin (Fig. 5c, d, comparing lane 3 vs lane 1). Importantly, upon knockdown of *LETN*, the full-length NPM1 also lost its rescuing effect on chromatin condensation (Fig. 5c, d, comparing lane 1 vs lane 2).

Similarly, another major function of NPM1, promoting rRNA synthesis, was also dependent on *LETN*. Knockdown of *LETN* significantly restrained the rescue of rRNA expression upon reintroducing NPM1 into the NPM1 knockout cells (Fig. 5e, f, comparing blue vs orange bars). ChIP analyses have demonstrated the direct binding of NPM1 to the genomic DNA around the rRNA gene promoter.^19,63,64^ Here, we further showed that *LETN* depletion significantly reduced the binding capacity of NPM1 to the rDNA promoter (Fig. 5g). In summary, *LETN* plays an indispensable role for the major functions of NPM1 in facilitating rRNA expression and chromatin condensation.

**Fig. 6:**
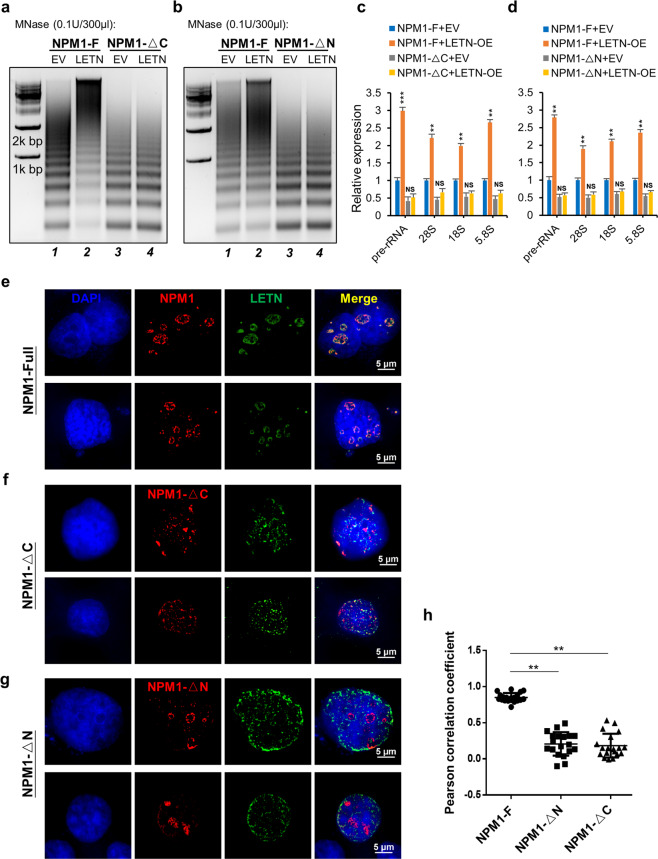
N- or C-terminal truncation of NPM1 blocked the major molecular functions of *LETN*. **a**–**d** Full-length NPM1 (NPM1-F), C-terminus-truncated NPM1 (NPM1-ΔC, panel **a**, **c**) or N-terminus-truncated NPM1 (NPM1-ΔN, panel **b**, **d**) was reintroduced into the NPM1^–/–^ HUH7 cells. The chromatin of these cells was partially digested with MNase (0.1 U/300 µL) upon overexpression of *LETN*. The resulted DNA fragments were visualized by gel electrophoresis (**a**, **b**). Relative expression levels of pre- and mature rRNAs were measured by RT-qPCR upon overexpression of *LETN* and normalized to the housekeeping gene beta-actin (**c**, **d**). The statistical significance levels were obtained by pair-wise comparisons between EV and *LETN*-OE (**a**, **b**). Data show means ± SD of three biological replicates. **e**–**g** SIM images showing the nucleus staining by DAPI (blue), mCherry-labeled full-length NPM1 (**e**), NPM1-ΔC (**f**), or NPM1-ΔN (**g**) (red), and MS2-tagged *LETN* marked by MS2-GFP (green) in the NPM1^–/–^ HUH7 cells. **h** The degrees of colocalization between *LETN* and the NPM1 proteins were quantified by the Pearson’s correlation coefficients (PCC). The maximal value of PCC (1.0) indicates perfect colocalization between two fluorescence signals in a cell, whereas PCC = 0 indicates no colocalization.

On the other hand, the molecular functions of *LETN* are executed mainly via NPM1. The data in Fig. 3 and Supplementary information, Figs. S14–S19 have shown that *LETN* knockdown led to disruptions of the cellular processes that are almost identical to those caused by NPM1 knockdown, whereas the overexpression of *LETN* did the opposite. Here, we further showed that the strong phenotypic consequences of *LETN* depletion (Fig. 5c–f) or overexpression (Fig. 6a–d) in HUH7 cells all depend on a fully functional NPM1 protein. Specifically, under the contexts of N- or C-terminally truncated NPM1, the cells were no longer responsive to knockdown or overexpression of the lncRNA *LETN* in terms of chromatin compactness (Fig. 5c, d for *LETN* knockdown and Fig. 6a, b for overexpression, all by comparing lane 3 vs lane 4) and rRNA expression levels (Fig. 5e, f for knockdown and Fig. 6c, d for overexpression, all by comparing the gray vs yellow bars).

Last, in the NPM1 knockout HUH7 cells, the re-expressed exogeneous NPM1 (mCherry-labeled) showed nice coaggregation with the exogenously expressed *LETN* in the nucleolus (Fig. 6e, h), which is well-expected given our previous results in Figs. 2 and 4. However, NPM1-ΔC was found all around the nucleus, but not concentrated in the nucleolus (Fig. 6f). This is because NPM1-ΔC lost its NoLS but retained its nuclear localization signal (NLS). Importantly, re-expression of NPM1-ΔC instead of the full-length protein blocked the nucleolar localization of the lncRNA *LETN* as well (Fig. 6f), and NPM1-ΔC showed almost no colocalization with *LETN* (Fig. 6f, h). As *LETN* binds to the C-terminus of NPM1 (Supplementary information, Fig. S11), the result above suggests that the nucleolar localization of *LETN* requires its binding with NPM1. In other words, the direct interaction between NPM1 and *LETN* is the cause, not a consequence, of *LETN*’s nucleolar localization.

NPM1-ΔN appeared to be enriched in the nucleolus (Fig. 6g), which is expected, as its C-terminal NoLS was intact. However, unlike the full-length NPM1, such partial aggregation of NPM1-ΔN was not accompanied by nucleolar colocalization with *LETN* (Fig. 6g, h). The N-terminal region mediated the oligomerization of NPM1, which is critical for its biological function of facilitating the structural organization and molecular functions of the nucleolus.^44,52,54–58^ Therefore, these data further confirmed that the nucleolar localization of *LETN* depends on a fully functional and oligomerized NPM1 in the nucleolus. Finally, it is worth noting that under the condition of *LETN* knockdown, the general distribution patterns of the two truncated proteins NPM1-ΔC and NPM1-ΔN were consistent to those in the control cells (Supplementary information, Fig. S22 compared to Fig. 6f, g).

In summary, for NPM1, its oligomerization and structured aggregation in the nucleolar GC requires the lncRNA *LETN*; and for *LETN*, its concentration in the nucleolus depends on the fully functional NPM1 protein. Such mutual dependency between NPM1 and *LETN* is crucial for their molecular functions in the nucleolus.

### Physiological relevance of the *LETN*–NPM1 interaction

**Fig. 7:**
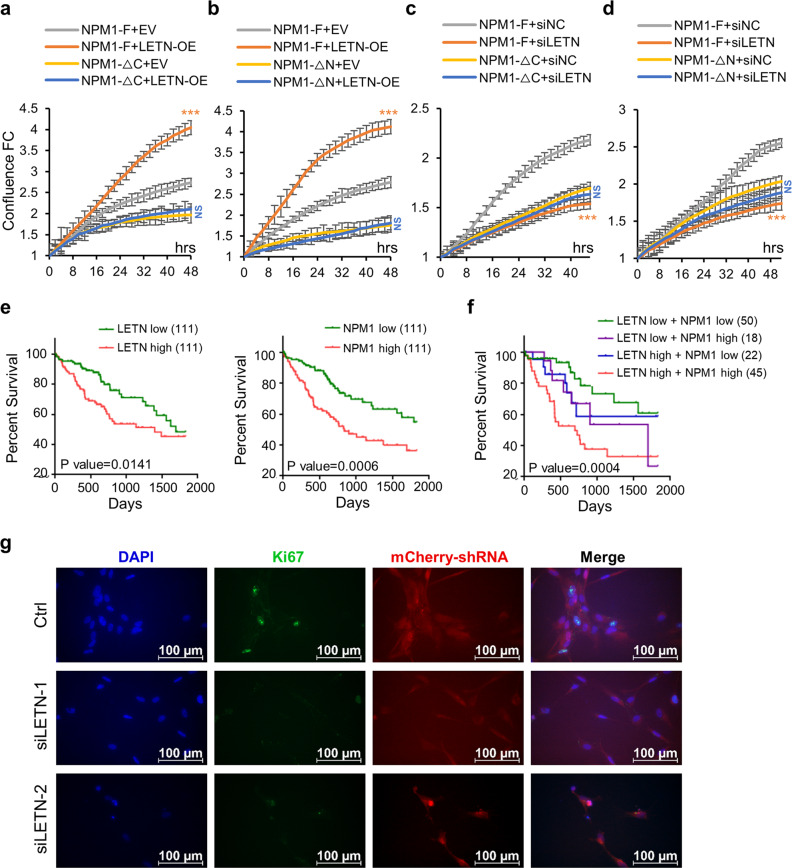
Physiological relevance of *LETN* in liver cancer and NPC proliferation. **a**–**d** Proliferation curves of NPM1^–/–^ HUH7 cells expressing full-length NPM1 (NPM1-F), NPM1-ΔC, or NPM1-ΔN, upon overexpression (**a**, **b**) or siRNA-mediated knockdown (**c**, **d**) of *LETN*. The statistical significance levels were obtained by pair-wise comparisons between EV and *LETN*-OE (**a**, **b**), or between siNC and si*LETN* (**c**, **d**). The error bars represent the ± SD of three biological replicates. **e**, **f** Liver cancer patients in TCGA were partitioned into subgroups according to the expression levels of *LETN* or NPM1 in the tumors. Kaplan–Meier survival curves were prepared for the subgroups of patients with *LETN* or NPM1 expression above the top 30th percentile or below the bottom 30th percentile (**e**). Next, intersections among the four groups above partitioned these patients further into four new subgroups (*LETN* low/high & NPM1 low/high). Kaplan–Meier survival curves for these subgroups were prepared (**f**). *P*-values were obtained with log-rank tests. **g** Proliferation signals shown by Ki67 staining in NPC cells derived from H9 ESC cells. The shRNA vectors used in this experiment were labeled with mCherry.

The results above have established a clear mutual dependency between the lncRNA *LETN* and the protein NPM1 in key molecular processes such as chromatin condensation and rRNA expression, which are crucial for fast proliferation of cells. As shown in Figs. 1 and 7a–d, overexpression or knockdown of *LETN* significantly accelerated or repressed cell proliferation, respectively. Such a strong phenotypic impact of *LETN* was only observed with the full-length NPM1 protein (Fig. 7a–d, comparing the gray vs orange curves) but not with the C- or N-terminally truncated NPM1 proteins (Fig. 7a–d, comparing the yellow vs blue curves). On the other hand, the rescue of cell proliferation by full-length NPM1 was only achieved with normal (Fig. 7a–d, comparing the gray vs yellow curves, *P*-values < 0.01) or overexpressed *LETN* (Fig. 7a, b, comparing the orange vs blue curves, *P*-values < 0.001), but not in the *LETN* knockdown cells (Fig. 7c, d, comparing the orange vs blue curves). Therefore, the *LETN*–NPM1 axis is critical for promoting and maintaining cancer cell proliferation.

Since cancer cells need hyperactive nucleoli to sustain fast cell proliferation, the nucleolar proteins would likely be associated with cancer patient prognosis. Indeed, the patients of liver cancer with lower expression levels of *LETN* or NPM1 had better prognosis (Fig. 7e). Furthermore, the patients with higher expression levels of both *LETN* and NPM1 showed the most aggressive cancer development that led to the worse prognosis (red curve in Fig. 7f), and by contrast, patients with lower expression levels of the both factors showed much better prognosis (green curve in Fig. 7f). Such association between the NPM1/*LETN* expressions and the patient survival supports the physiological relevance of the NPM1–*LETN* axis via its involvement in promoting the nucleolar functions.

In addition to the indispensable role in tumorigenesis,^29–31^ NPM1 has also been shown to be essential for embryonic development, especially in the forebrain.^29^ Given the dependency of NPM1 function on the lncRNA *LETN*, we further asked whether *LETN* is also critical for brain development. The expression of *LETN* is low in most adult human organs, and its relatively high expression was seen mainly during early gestational age (GA), especially in the fetal brain and cerebellum around GA weeks 7–10^32^ (Supplementary information, Fig. S1a). This is the stage of embryogenesis and early fetal development when the NPCs undergo fast proliferation.^65^ Indeed, we confirmed that the induction of human embryonic stem cell (ESC) H9 to NPCs coincides with increased expression of *LETN* (Supplementary information, Fig. S23), which was consistent with the data from another published study.^66^ Interestingly, upon *LETN* knockdown, the H9-induced NPCs showed largely repressed proliferation signal of Ki67 (Fig. 7g). Therefore, the lncRNA *LETN* is not only indispensable for tumor cell proliferation but also very likely to be relevant in normal organ development, for example, in fetal brain development.

### *LETN*–NPM1 interaction is associated with evolutionarily new variations of NPM1

As a critical structural and functional nucleolar protein, NPM1 is highly conserved across organisms. However, the sequence of *LETN*, like many other human lncRNA species, is not well conserved (Supplementary information, Fig. S24a). Similar genomic sequences of *LETN* can be found in multiple primates such as chimpanzee, gorilla, orangutan, etc., but not in most of the other vertebrates including the model animals mouse and rat (Supplementary information, Fig. S24a). Therefore, it is worth investigating why the highly conserved NPM1 in human cells depends on the lncRNA *LETN* that is absent in other species such as mouse.

After NPM1 knockdown in HUH7 cells, expression rescue with mouse or human NPM1 largely restored the cell proliferation rate (Supplementary information, Fig. S24b) and the rRNA expressions (Supplementary information, Fig. S24c). Interestingly, while the rescue effects of human NPM1 depend on the expression of *LETN* (Figs. 5–7; Supplementary information, Fig. S24b, c), mouse NPM1 restored the proliferation rates and the rRNA expressions equally with and without *LETN* (Supplementary information, Fig. S24b, c). These results suggest that although the NPM1 protein sequences are largely conserved across human and mouse, there is still a critical difference between these two versions of NPM1 in terms of their dependency on *LETN*.

**Fig. 8:**
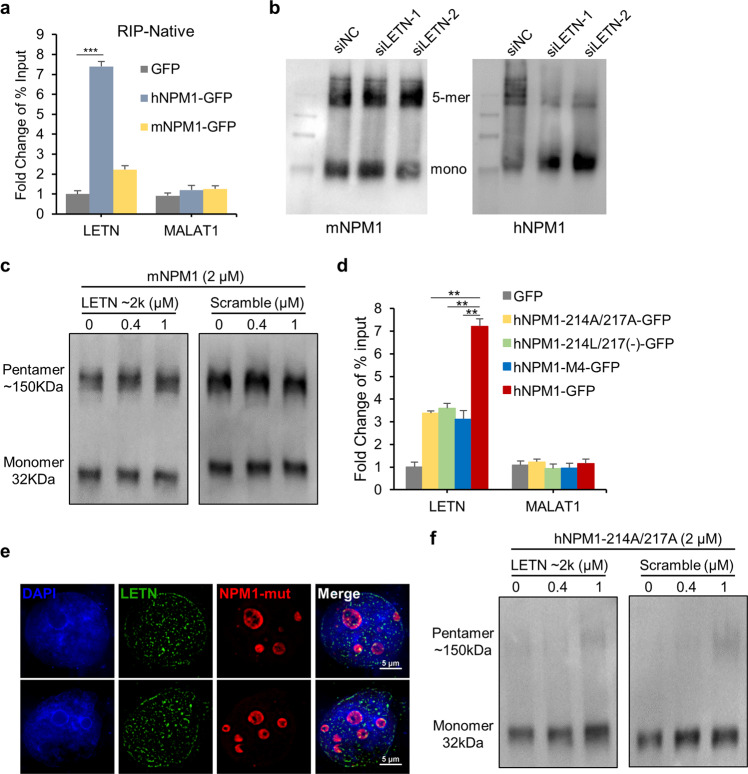
Differences between human and mouse NPM1 in the interactions with *LETN*. **a** GFP-tagged human or mouse NPM1 was reintroduced into the NPM1^–/–^ HUH7 cells. qPCR of *LETN* and *MALAT1* was performed after native cross-linking GFP-RIP. *MALAT1* was used as a negative control. The error bars represent the ± SD of three biological replicates. **b** Human or mouse NPM1 was reintroduced into NPM1^–/–^ HUH7 cells. Native PAGE was used to show the oligomeric and monomeric NPM1 proteins under the conditions of *LETN* knockdown. **c** In vitro assays with purified mouse NPM1 monomeric protein, which was incubated for about 40 min with *LETN* fragment of the first ~2000 nt or scrambled RNA with a similar length. Images of native PAGE with anti-NPM1 show the oligomeric and monomeric NPM1 proteins. **d** GFP-tagged human wild-type or mutant NPM1 at the two residues (214 and 217) or at the three residues altogether (M4) was reintroduced into NPM1^–/–^ HUH7 cells. qPCR of *LETN* and *MALAT1* was performed after native cross-linking GFP-RIP with the cells. *MALAT1* was used as a negative control. The error bars represent the ± SD of three biological replicates. **e** SIM images showing the nucleus staining by DAPI (blue), mCherry-labeled NPM1-214A/217A mutant protein and *LETN* (green) in the NPM1^–/–^ HUH7 cells. **f** In vitro polymerization with purified human NPM1-214A/217A mutant protein, which was incubated for about 40 min with *LETN* fragment of the first ~2000 nt or scrambled RNA with a similar length. Images of native PAGE with anti-NPM1 show the oligomeric and monomeric NPM1 proteins.

Indeed, to our surprise, mouse NPM1, which was expressed in the NPM1^–/–^ HUH7 cells, weakly binds to *LETN*, if not none at all (Fig. 8a), while similarly restored human NPM1 showed strong binding with *LETN* (Fig. 8a, also supported by other results above). Interestingly, under the similar context of NPM1 restoration in the NPM1^–/–^ HUH7 cells, pentamerization of mouse NPM1 was not affected by *LETN* knockdown at all, whereas human NPM1 largely shifted towards the monomeric states upon *LETN* knockdown (Fig. 8b). In addition to the in vivo experiments above, in vitro assays also showed that the purified mouse NPM1 proteins form pentamers easily without *LETN* and that the addition of *LETN* did not further promote the pentamerization (Fig. 8c). Compared to the results in Fig. 4d, this again exhibits an obvious difference between the human and mouse NPM1 proteins.

The N-terminal region of NPM1 (amino acids 1–121) is exactly the same between human and mouse, whereas the middle region (122–189) harbors a few mutations (Supplementary information, Fig. S25a). This region, also known as an intrinsically disordered region, is highly enriched by acidic residues and therefore responsible for the binding of NPM1 to histones.^51,53^ Normally, this function is unlikely to be affected by the few variations within this region. Importantly, we have already shown that binding with *LETN* takes place in the C-terminal part of NPM1 (residues 190–294, Supplementary information, Fig. S11). Compared to the mouse NPM1, this C-terminal region of human NPM1 harbors mutations at four residues, i.e., 190 (Val to Ala), 196 (Val to Ile), 214 (Leu to Ser), and 217 (Ser insertion) (Supplementary information, Fig. S25a). Due to the similar physicochemical properties between Val and Ala/Ile, the first two mutations are unlikely to result in significant functional changes of the protein. Therefore, we suspected that the newly acquired functional dependency of human NPM1 on *LETN* could be attributed to these four residues, most likely to the latter two Ser residues.

In the NPM1 rescue experiment with NPM1^–/–^ HUH7 cells, converting any of these four residues of human NPM1 to the mouse version only slightly reduced its binding affinity with *LETN*, whereas combinatory mutation of all four of them largely abolished its binding with *LETN* (Supplementary information, Fig. S25b). Note that the same level of binding blockage with *LETN* was achieved by mutating just the two Ser residues 214 and 217, to their mouse homologs or to the non-phosphorylatable mimic Ala (Fig. 8d). In vitro assays also confirmed that the human NPM1-214A/217A mutant proteins almost completely lost their binding capacity with *LETN* (Supplementary information, Fig. S25c). Consistently, as its NoLS signals are intact, the human NPM1-214A/217A mutant proteins were enriched in the nucleolus (Fig. 8e), whereas *LETN* was found spread in the nucleus, rather than being enriched in the nucleolus like it was in the cells with wild-type human NPM1 (Fig. 8e, comparing to Figs. 2 and 6). Furthermore, in vitro NPM1 polymerization assays showed that the human NPM1 mutant (hNPM1-214A/217A) does not form pentamers with or without *LETN* (Fig. 8f). Therefore, the two Ser sites are critical for the interaction between *LETN* and NPM1, which controls the pentamerization and the molecular functions of NPM1.

From the other way around, conversion of the mouse NPM1 to the human homologs at the two residues or the four residues altogether largely restored its binding with *LETN* to the same level (Supplementary information, Fig. S25d). In vitro NPM1 polymerization assays showed that the mutation of mouse NPM1 (mNPM1-214S/217S) blocked the protein pentamerization (Supplementary information, Fig. S25e). However, *LETN* strongly reactivated the pentamerization of the mouse NPM1 mutant (mNPM1-214S/217S) (Supplementary information, Fig. S25e). Together, the results above suggest that these two Ser residues are predominately responsible for the NPM1–*LETN* interaction in human. Indeed, the combination of these two Ser residues is only found in primates, which is well in line with the conservation patterns of the *LETN*-like sequences (Supplementary information, Fig. S26). Therefore, it appears that the *LETN*–NPM1 interaction is associated with the evolutionarily new variations of NPM1 and the coordinated emergence of the lncRNA. Such a protein–lncRNA interaction renders an additional yet critical layer of regulation for the nucleolar functions that sustain fast cell proliferation.

## Discussion

Great efforts have been dedicated to functional annotations of the non-coding transcriptomes in human and other species.^5–7,9,10,32,67^ In general, the scale of the non-coding genome is associated with the complexity of the species.^2^ Hundreds of lncRNAs are associated with a large variety of normal biological activities and disease processes.^6,7^ Studies have indicated that many of these lncRNAs function as fine-tuners rather than key players in processes such as development or disease.^5,9,10,67^ In the present study, we found that a previously uncharacterized lncRNA, which we renamed as *LETN*, plays an indispensable role in facilitating the molecular functions of NPM1 that shape the organized nucleolus structure and sustain cell proliferation. There are several open reading frame (ORF) candidates in the *LETN* sequence. However, based on bioinformatic assessments of their coding potentials with CPAT^68^ and validations with several mass spectrometry datasets and ribosome profiling data^69^ from tissues of liver, liver tumor, and cancer cell lines, we did not see any support of peptide coded by any of these ORFs. Finally, as shown in our manuscript, *LETN* is primarily located in the nucleolus. Therefore, *LETN* should mainly function as a non-coding RNA and it is unlikely to produce any peptide or protein.

NPM1 is a well-studied nucleolar chaperone protein that is essential for the assembly of the nucleolus.^19,20,24^ It is highly abundant and has been shown to play critical roles in key processes, such as ribosome biogenesis and chromatin condensation.^19,20,24–28^ Strikingly, these major functions of NPM1 all depend on the lncRNA *LETN*. On the other hand, the physiological relevance of *LETN* was mainly mediated by the highly powerful protein NPM1. Eventually, we showed that this mutual interaction between *LETN* and NPM1 is essential for the proliferation of cancer cells and potentially NPCs. Therefore, *LETN* is a rare case of the lncRNAs with fundamental biological activities and high physiological relevance.

The nucleolus is a crucial organelle for fundamental processes such as rDNA transcription and ribosome biogenesis.^11^ Previously, several lncRNAs have been reported to facilitate major functions of the nucleolus. For example, the lncRNA PAPAS, an antisense transcript to pre-rRNA functioning *in cis*, can help recruit CHD4/NuRD to the rDNA promoter and repress rDNA transcription at elevated temperature.^14^ Upregulated PAPAS in quiescent cells results in increased H4K20me3 at rDNAs and repressed transcription.^15^ The lncRNA SLERT is another example of nucleolar lncRNAs, which acts *in trans* and derepresses Pol I transcription of rDNA by binding to DDX21 and loosening the DDX21 ring.^16^ Another lncRNA, HOXB-AS3, was reported to guide the ErbB3-binding protein 1 (EBP1) to the rDNA, therefore promoting the rRNA transcription.^17^ While being influential on the nucleolar functions, none of these nucleolar lncRNAs has been reported to be impactful on the structure of the nucleolus itself. From this perspective, *LETN* is unique, as it is essential for the normal organized structure and multiple major functions of the nucleolus, which is supported by the strong phenotypic responses of cancer cells and NPCs to the repression of *LETN*.

The nucleolus has been well recognized as a typical membraneless organelle composed of different compartments and formed by liquid–liquid phase separation (LLPS).^22,70–72^ The oligomerized NPM1 protein, as pentamers, exhibits multivalency in binding with arginine-rich linear motifs (R-motifs) and rRNA, which enables heterotypic LLPS.^20^ The pentameric ring of NPM1 also undergoes homotypic assembly via LLPS.^21^ These multiple LLPS processes have been proposed to be simultaneously operational and essential for the dynamic liquid-like features of the nucleolus.^20,21^ Furthermore, the different surface tensions of the liquid droplets composed of different proteins define the core-shell structure of the nucleoli, of which the GC is enriched with NPM1.^22^ This implies that the organization of the nucleolus is a dynamic process and that the nucleoli could have multiple states with potentially differential functional activities. First, the sizes of the nucleoli have been shown to be well related to the rates of cancer cell proliferation.^73^ In addition, while the well-recognized round-shaped nucleoli have been frequently shown in literature, other reticulated nucleoli have also been reported,^74,75^ mostly in the normal non-proliferative cells. Such distorted nucleoli appear quite similar to the nucleoli morphology upon *LETN* knockdown (Figs. 3a, 4e, f) or NPM1 knockdown^23,50^ (Fig. 3a). Therefore, based on the results of our study, we propose that NPM1–*LETN* plays a critical role in ensuring the hyperactive nucleolar state, to which cancer is known to be ‘addicted’ for rapid growth.^76^

Focusing on the nucleolar GC morphology, we noted that the SIM images showing the IF staining of the endogenous NPM1 and NCL (Fig. 4e, f; Supplementary information, Fig. S19) do not appear perfectly consistent with the time-lapse live cell images in Fig. 2e, Supplementary information, Fig. S13b and Videos S1–S5. The SIM images have leveraged the high resolution to illustrate the fluorescence distributions on a thin cross-section of the nucleolus. Therefore, the peripheral mass (i.e., GC) of the nucleolus presents a nearly ring-shaped distribution pattern on a single SIM image. The 3D structures of the nucleoli reconstructed with multiple SIM images at different depths on the *z*-axis show the nucleolar GC much clearer (Supplementary information, Videos S6–S9). In addition, although our IF staining experiments have been extensively optimized, the possibility of reduced accessibility of the intra-nucleolar proteins by the antibodies cannot be completely ruled out. However, this possibility does not dampen the observation that knockdown of *LETN* perturbed the nucleolar morphology. On the other hand, the time-lapse microscopy of live cells just shows the overall colocalization between *LETN* and the mCherry-labeled NPM1. Although the time-lapse images occasionally captured the ring-shaped NPM1 and *LETN* distribution patterns as well, their resolution and sensitivity were not high enough to illustrate the detailed distribution of NPM1 or *LETN* on a single cross-section of the nucleolus.

Importantly, it is the NPM1 pentamer, instead of the monomer, that forms the fundamental building blocks of the LLPS processes. The studies above illustrated that the purified NPM1 forms pentamers and undergoes LLPS, and we also saw predominance of the pentamers formed by purified NPM1 in vitro. These data suggest that the NPM1 pentamers, once formed, are stable in vitro without *LETN*. However, we have showed that oligomerization of NPM1 in vivo depends on the lncRNA *LETN*. Furthermore, after breaking most of the oligomers into monomers by sonication (Fig. 4d), *LETN*, but not scrambled RNA, greatly facilitated re-oligomerization of the monomeric NPM1 in vitro (Fig. 4d). Therefore, based on the results above altogether, we propose the following potential scenario. The lncRNA *LETN*, being recruited by NPM1 into the nucleoli, facilitates the formation of the NPM1 pentamer, which then takes a central position in shaping the nucleolus and sustaining the nucleolar functions. Due to the high abundance of NPM1 in the nucleolus, the lncRNA is restrained in the nucleolus and reused for many rounds of NPM1 pentamerization. In other words, the *LETN*–NPM1 interaction is a one-to-many type, and this explains why the relatively small number of the *LETN* molecule play such a critical role in facilitating the pentamerization of NPM1, which is much more abundant than *LETN*.

Finally, the current study has been focused on the nucleolar functions depending on NPM1 and *LETN*, as both factors are highly enriched in the nucleoli. However, some results also indicated potentially extra-nucleolar functions of NPM1 and *LETN*. For example, knockdown of *LETN* or NPM1 seems to have a rather global effect on the chromatin condensation (Fig. 3c), which is unlikely to be just limited in the nucleolus. In fact, although NPM1 has been best-characterized as a nucleolar protein, it also carries out various functions outside of the nucleoli (for example^77^). Interestingly, previous studies have identified other NPM1-interacting lncRNAs that regulate the extra-nucleolar functions of NPM1. For example, SAMD12-AS1, activated by HBV-encoded HBx, titrates away NPM1 from the E3 ligase HDM2, which resulted in increased p53 degradation.^78^ In the present study, we have also observed sparse distributions of NPM1 occasionally in the nucleoplasm, and very interestingly, *LETN* was found to colocalize with NPM1 even outside of the nucleoli (for example Fig. 6e). Therefore, we suspect that these extra functions of NPM1 outside of the nucleolus are very likely to be related to its colocalization with *LETN* as well. Such extra-nucleolus functions of the NPM1–*LETN* axis is certainly worth clarifying in the future.

## Materials and methods

### Survey of functional lncRNAs in cancers

We adapted the conceptual design of the Modulator Inference using the Network Dynamics (MINDy) algorithm^79^ and developed a data-mining pipeline to search for the lncRNAs that exhibited modulatory functions on the gene regulatory circuits from transcription factors to their target genes. Previously, we have used the same methodology to similarly infer the DNA methylation sites that modulate the transcriptional regulatory circuits,^80^ and to identify the transcription factors whose activities on the target gene expressions depend on the level of a specific lncRNA, *NEAT1*.^81^

The large-scale mRNA and lncRNA expression data of tumor samples in The Cancer Genome Atlas (TCGA) was used for the analysis, which was done in a cancer type-specific manner for 20 major cancers in TCGA (manuscript under review). The present study was based on the results in liver cancer. Specifically, in liver cancer, the lncRNA ENST00000564237.1 (RP11-196G18.22) was predicted to modulate the largest number of transcriptional regulation circuits, i.e., pairs of transcription factors and their target genes, suggesting that this lncRNA potentially serves as a powerful determining factor of the gene expression programs in liver tumors.

### Analysis of the DNA copy number and RNA expression data from TCGA

The pan-cancer copy number variation (CNV) data in TCGA were downloaded from UCSC Xena (http://xena.ucsc.edu/). GISTIC2 threshold method was applied using the TCGA FIREHOSE pipeline to produce gene-level copy number estimates. Subsequently, as done previously in literature,^82,83^ the CNV values were partitioned into five groups: −2, −1, 0, 1, 2, representing homozygous deletion, single copy deletion, diploid normal copy, low-level copy number amplification, or high-level copy number amplification, respectively.

As previously described,^84^ RNA-seq V2 data (level 3) were used for *LETN* expression profiles in tumor and adjacent normal samples from 22 cancer types in TCGA. These are read counts of genes and have been already processed and normalized across all the samples of each cancer type by TCGA with the upper quantile normalization method.

### Cell culture

Cell lines were purchased from American Type Culture Collection (ATCC) and cultured in a humidified incubator with 5% CO_2_ at 37 °C. HUH7 and HCC827 cells were cultured in Dulbecco’s modified Eagle medium (high glucose, Corning). PC3 cells were cultured in Roswell Park Memorial Institute 1640 (RPMI-1640, Corning), and DU145 cells were cultured in Eagle’s Minimum Essential Medium (MEM, Corning). All the media were supplemented with 10% fetal bovine serum (HyClone).

### Estimation of *LETN* RNA copy numbers per cell

Different concentrations of RNA spike-in was added into the total RNA extracted from 30,000 cells with TRIzol. The RNA samples were then subjected to reverse transcription followed by qPCR analysis. The serial dilution of RNA spike-in was used to generate the standard curve, which shows the Ct values for the RNA copy numbers in 30,000 cells. The copy number of *LETN* per cell was then estimated by fitting its Ct value on to the standard curve.

### 5′- and 3′-RACE

The lncRNA *LETN* full length containing the transcriptional initiation and termination sites was confirmed by 5′- and 3′-RACE using the SMARTer RACE 5′/3′ Kit (Takara Bio USA, Cat. Nos. 634858) by following the manufacturer’s protocol. The gene-specific primers (GSPs) were designed for 5′-RACE (5′TCACCATTACCGCTACATAGTACG3′) and 3′-RACE (5′GAGACATGCCACCATATTTAGG3′) PCR.

### Vector construction, small interfering RNA (siRNA) synthesis and transfection

Full-length *LETN* was PCR-amplified by Q5 High-Fidelity DNA Polymerase (NEB) from genomic DNA of HUH7 cells. The PCR product was cloned into the *Eco*RI and *Not*I sites of the pcDNA3.1 vector. To generate the pcDNA3.1-MS2 and pcDNA3.1-*LETN*-MS2 vectors, the MS2-12× fragment was PCR-amplified by Q5 High-Fidelity DNA Polymerase (NEB) from pSL-MS2-12× (Addgene) and cloned into the *Not*I and *Xba*I sites of pcDNA3.1 and pcDNA3.1-*LETN*, respectively.

The cDNA of the NPM1 fragments (NPM1.1, NPM1.2, NPM1.3 as shown in Supplementary information, Fig. S11), truncated NPM1 (NPM1-ΔN (amino acids 122–294 remaining) and NPM1-ΔC (amino acids 1–189 remaining)), or NPM1 full-length were PCR-amplified by Q5 High-Fidelity DNA Polymerase (NEB) and cloned into the *Eco*RI and *Not*I sites of pLV-mCherry or pLV-GFP vectors.

The siRNAs specifically targeting *LETN*, NPM1, B2M and LMNA, and non-targeting control siRNA were synthesized by GenePharma. siB2M and siLMNA served as negative controls. ASO specifically targeting *LETN* and control ASO-NC were synthesized by RiboBio. Cells were transfected with the plasmids, siRNAs, or ASO using Lipofectamine 2000 or Lipofectamine RNAiMAX Reagent (Invitrogen), respectively, following the manufacturer’s protocol.

**Table:** 

Oligos	Sense 5′-3′	Antisense 5′-3′
si*LETN*-1	GCUGUCUCCAUGUCUUCUU	AAGAAGACAUGGAGACAGC
si*LETN*-2	GCUCUCUGCUCAAGUAUUA	UAAUACUUGAGCAGAGAGC
siNPM1-1	CCGACAAAGAUUAUCACUU	AAGUGAUAAUCUUUGUCGG
siNPM1-2	GGAAGAUGCAGAGUCAGAA	UUCUGACUCUGCAUCUUCC
siB2M	UACAAGAGAUAGAAAGACCAG	CUGGUCUUUCUAUCUCUUGUA
siLMNA	AUCUCAUCCUGAAGUUGCUUC	GAAGCAACUUCAGGAUGAGAU
siNC	ACGUGACACGUUCGGAGAA	UUCUCCGAACGUGUCACGU
ASO-*LETN*	GGCUGUCUCCAUGUCUUCUU	AAGAAGACAUGGAGACAGCC

### Lentivirus production and construction of stable cell lines

For overexpression, the full-length *LETN* was cloned into the *Eco*RI and *Sgr*AI sites of pLV-mCherry-N vector, named as *LETN*-OE. For shRNA-mediated knockdown, two pairs of cDNA oligonucleotides targeting *LETN* were designed and synthesized. After annealing, double-strand oligonucleotides were inserted into pLVshRNA-mCherry(2A) puro vector, named as sh*LETN*-1 and sh*LETN*-2. A scrambled shRNA was used as negative control.

The individual lentiviral expression vector was mixed with the packaging plasmid Δ8.9 and VSVG. The plasmids were co-transfected into HEK293T cells with Lipofectamine 2000 (Invitrogen). After 48 h, the medium was collected and passed through a 0.45 µm filter. The cells were infected by the medium with 8 µg/mL of polybrene. After 24 h of infection, the medium was removed, and the cells were selected with puromycin.

**Table:** 

shRNA	Sense 5′-3′	Antisense 5′-3′
sh*LETN*-1	GGACTTCCAGTTAAGCCAATT	AATTGGCTTAACTGGAAGTCC
sh*LETN*-2	GGCTGTCTCCATGTCTTCTTA	TAAGAAGACATGGAGACAGCC
shNC	ACGTGACACGTTCGGAGAAA	TTTCTCCGAACGTGTCACGT

### Knockout of *LETN* or NPM1 by CRISPR/Cas9

The sgRNAs targeting *LETN* or NPM1 were individually inserted into PX458 plasmid (Addgene), which also expresses GFP. The plasmids were transfected into HUH7 cells with Lipofectamine 2000 reagent (Invitrogen). The positive cells which expressed GFP protein were screened by FACS and the knockout efficiency was detected by RT-qPCR.

**Table:** 

sgRNA	Sequence 5′-3′
sg*LETN*-1	TCAAATTTCAGTCGGAACTC
sg*LETN*-2	GAGACGATATGCTACGGGTG
sgNPM1-1	GCGCAGGACGGCTACGGTAC
sgNPM1-2	GTTAACTATCATCAGGAGGT
Scramble sgRNA-1	GAACGTTGGCACTACTTCAC
Scramble sgRNA-2	GCGCCTTAAGAGTACTCATC

### Cell proliferation and colony formation assays

After different types of gene perturbations, the cells were cultured in 48-well plates, at the starting density of 20,000 cells per well. The IncuCyte live-cell imaging and analysis system (ESSEN Bioscience) was used to monitor real-time cell proliferation and morphology changes. Cell proliferation was quantified by measuring the occupied area (% confluence) in the cell images over time. The proliferation curves were made with confluence fold change (FC) at different time points in relative to the confluence at time 0.

For colony formation assays, 1000 cells were seeded in the 6-well plates and incubated with normal growth medium for 14 days. Then cells were fixed and stained with 0.5% crystal violet for 15 min. Lastly, the colonies were imaged via a camera or a microscope. For the anchorage-independent colony formation assay, the cells were collected, resuspended in culturing media containing 10% FBS and 0.3% agarose (Amresco), and then seeded on top of 0.6% agarose gel containing 10% FBS in six-well plates. The cells were cultured in regular media for 3–4 weeks, and the colonies were stained with crystal violet and photographed.

### In vivo xenograft assay

Xenograft animal models were established with male athymic BALB/c nude mice (6 weeks old). First, cells (3 million for the *LETN* knockdown experiment or 1 million for overexpression) were injected into the left or the right flanks of mice subcutaneously. Tumors were allowed to grow for 4–5 weeks. The tumor volume was measured every 7 days and calculated as 0.5 × a (long diameter) × b^2^ (short diameter). At the end, the mice were sacrificed, and the tumors were isolated, photographed and weighed. All the mouse studies have been approved by the Institutional Animal Care and Use Committee at Tsinghua University.

### RNA extraction and real-time qPCR analysis

Total RNA was isolated using the TRIzol reagent (Invitrogen) following the manufacturer’s instructions. The first-strand cDNA was generated using a High-Capacity cDNA Reverse Transcription Kit (Invitrogen, 4368814) and diluted 1:10 in nuclease-free water to use as a template for qPCR. Real-time qPCR was carried out with SYBR Green Master MIX (Invitrogen) and the gene-specific primers were shown as follows. The gene expression values in relative to the house-keeping genes GAPDH or beta-actin were calculated by the Ct differences (ΔCt), and the fold change of the gene expression in relative to the control sample were calculated by the difference between the ΔCt values (ΔΔC).

**Table:** 

Gene ID	Forward primer 5′-3′	Reverse primer 5′-3′
*LETN*-1	GGGTCTACCTGTGAACTGTGA	GGAAACACATGTGGCAGCAC
*LETN*-2	TGGTTTCTGGCAGCATAACT	AGCCTGGGCAACAAGAGTGA
*NPM1*-N	TTCGGTTGTGAACTAAAGGC	CAAGGGAAACCGTTGGCTGT
*NPM1*-C	TCTGTAGAAGACATTAAAGCAAA	AATAGCCTCTTGGTCAGTCAT
Pre-rRNA	GCCTTCTCTAGCGATCTGAGAG	CCATAACGGAGGCAGAGACA
18S rRNA	TCCTTTGGTCGCTCGCTCCT	GATCTGATAAATGCACGCATCCC
5.8S rRNA	ACTCGGCTCGTGCGTC	GCGACGCTCAGACAGG
28S rRNA	GCGGGTAAACGGCGGGAGTA	TTGGCTGTGGTTTCGCTGGAT
Spike in	TAAACAGGATACCCGTCATCTGAAAGT	ATCGCCGTCGCTGATGGAGT
*NEAT1*	TTCACCTGCTCTGGCTCTTG	GCCAGGCACCGTGTTATACT
*HOTTIP*	CCTAAAGCCACGCTTCTTTG	TGCAGGCTGGAGATCCTACT
*HOTAIR*	GGGGCTTCCTTGCTCTTCTTATC	GGTAGAAAAAGCAACCACGAAGC
*SLERT*	TTAGTCAGCTCAGGCCCAGT	AAGTGCTCCACCAACTCCAG
*NESTIN*	GCGGCTGCGGGCTACTGAAA	CCAGGAGGGTCCTGTACGTGGC
*NONOG*	CAATGGTGTGACGCAGAAGG	TGCACCAGGTCTGAGTGTTC
*GAPDH*	GGTCACCAGGGCTGCTTTTA	TTCCCGTTCTCAGCCTTGAC
*ACTB* (beta-actin)	TGGACATCCGCAAAGACCTG	CCGATCCACACGGAGTACTT
*MALAT1*	GCTCTGTGGTGTGGGATTGA	GTGGCAAAATGGCGGACTTT

### Northern blotting

For the northern blot assay, 5 µg total RNA was resolved by agarose gel electrophoresis, followed by transferring to a nylon membrane. The membrane was then subjected to UV crosslinking (120 mJ/cm^2^) immediately, followed by pre-hybridization for 1 h at 68 °C, and then hybridization with DIG labeled probes for 16 h at 68 °C. After blocking with the blocking buffer (Roche, 11585762001), the membrane was incubated with anti-DIG-AP antibody. The antisense probe was synthesized with the DIG RNA labeling Mix (Roche) and SP6 polymerase (Thermo). The antisense probe sequences are TCAGCTCTCTGCTCAAGTA and ACATGTTAAGCATACTGCGG for *LETN*, GAGAACGCCTGACACGCAGG and GCTGACACGCTGTCCTCT for Pre-rRNA, and CCGATCCACACGGAGTACTT and AGAAAATCTGGCACCACACC for beta-actin.

### Labeling of newly synthesized nascent RNAs

To capture the newly synthesized nascent RNAs, 0.2 mM EU was added to the culture medium for 24 h. EU-incorporated RNAs were biotinylated and captured by using the Click-iT Nascent RNA Capture Kit (Invitrogen, MP10365) in accordance with the manufacturer’s instructions.

### Western blotting and native polyacrylamide gels

Cells were harvested and lysed with RIPA lysis buffer (Solarbio) supplemented with 1 mM PMSF and protease inhibitor cocktail (Roche, 4693124001). Lysate was quantified using the BCA protein assay (Pierce). The cell lysate containing 30 µg proteins was prepared in a 1× SDS buffer and heat-denatured. Then sample was subjected to 10% SDS-PAGE, followed by transferring to a NC membrane. After blocking using 3% BSA, the membrane was incubated with the primary and secondary antibodies. The protein bands were visualized and quantified with the SuperSignal West Pico Chemiluminescent HRP substrate (Thermo) and imaged on the Molecular Imager ChemiDox XRS System from Bio-Rad. Anti-NPM1 (1:2000, ab10530), anti-GFP (1:2000, ab290), anti-Histone H2A (1:2000, EPR17470, ab177308) and anti-Histone H2B (1:2000, EP957Y, ab52599) were purchased from Abcam, anti-Histone H3 (1:2000, 17168-1-AP), anti-Histone H4 (1:1000, 16047-1-AP) and anti-GAPDH (1:5000, 60006-1-lg) were purchased from Proteintech. Secondary anti-rabbit and anti-mouse were purchased from Pierce Biotechnology.

For Native gel electrophoresis, the cells were lysed with RIPA buffer without SDS, supplemented with 1 mM PMSF and protease inhibitor cocktail (Roche, 4693124001), followed by short-lived sonication. The cell lysates were mixed with 4× Protein Native PAGE Loading Buffer (TaKaRa, 9175) without heating and then loaded into native PAGE gels without SDS. The other procedures were the same as regular protein gel electrophoresis.

### Isolation of cytoplasmic and nuclear RNA

The cells were harvested and lysed with 200 µL lysis buffer (50 mM Tris, pH 8.0, 140 mM NaCl, 1.5 mM MgCl_2_, 0.5% NP-40, 2 mM Ribonucleoside Vanadyl Complex). After incubation on ice for 10 min, the lysate was centrifuged at 1000 rpm for 3 min at 4 °C. The supernatant was enriched by cytoplasmic fraction, and the nuclear fraction was in the pellet. The pellet (nuclear fraction) was collected and resuspended in lysis buffer (without NP-40).

For nucleolus isolation, the pellet (nuclear fraction) was dissolved with resuspension buffer I (340 mM sucrose and 5 mM MgCl_2_), followed by sonication until the pellet was completely resolved. The equal volume of resuspension buffer II (880 mM sucrose and 5 mM MgCl_2_) was added, followed by centrifugation at 2000 rpm for 20 min at 4 °C. The supernatant was enriched by the nucleoplasmic fraction, and the nucleolus fraction was in the pellet. Finally, TRIzol was used to extract RNA from the cytoplasmic, nucleoplasmic and nucleolus fractions.^16,85^

### Chromatin condensation assay by MNase digestion

200,000 cells were harvested and lysed with 2 mL lysis buffer I (50 mM HEPES with the pH at 7.6, 140 mM NaCl, 1 mM EDTA, 10% glycerol, 0.5% NP-40, and 0.25% Triton X-100) for 30 min at 4 °C. The lysate was centrifuged at 1000 rpm for 5 min, and the supernatant was discarded. The pellet (chromatin fraction) was washed with lysis buffer II (10 mM Tris-Cl at pH 8, 200 mM NaCl, 1 mM EDTA, 0.5 mM EGTA) and then 10 mM Tris-Cl buffer at pH 7.5. The chromatin pellet was dissolved with MNase digestion buffer (10 mM Tris-Cl at pH 7.5, 50 mM NaCl, 5 mM MgCl_2_, 1 mM CaCl_2_, 0.075% NP-40) containing MNase (concentrations indicated on the figures) for 5 min at 37 °C on a rotating platform. The MNase reaction was stopped by adding the Stop solution (5% SDS and 50 mM EDTA). The DNA fragments were isolated by PCI (Phenol:Chloroform:Isoamyl Alcohol (25:24:1)) extraction and analyzed on 1% agarose gels.

### RNA sequencing and differential expression analysis

Total RNA was extracted with TRIzol reagent, following the manufacturer’s protocol. The Ribozero Kit (Epicentre) was used to remove ribosomal RNA. The ribosomal RNA-depleted RNA was used for sequencing library preparation with a NEB Next Ultra Directional RNA Library Prep Kit (NEB). Next-generation sequencing was performed on the Illumina HiSeq X Ten system.

Truseq library 2 × 150 reads from total RNA sequencing were first pre-processed using Cutadapt to remove adapters and trim low-quality bases from 5′ and/or 3′ ends. After discarding reads shorter than 20 bp, paired-end reads were mapped to the hg19 genome using STAR (v1.2.15), with ENSEMBLE V75 reference annotation and the default parameters. Differential gene expression analyses were performed with the R package DEseq2. In the results, the genes with |log_2_Fold change| > 1 and *P*-value less than 10^−5^ were deemed as significantly upregulated or downregulated and used for downstream analyses.

### Functional annotations and gene set enrichment analysis

For the various gene sets from different analyses, GO enrichment analysis was conducted using the Metascape tool. The GO terms with *P*-values < 0.001 were selected and visualized by clusterProfiler. Each GO term was color-coded according to the *P*-value (−log_10_) of the enrichment. The size of each node is proportional to the number of genes belonging to the GO term.

### RNA fluorescence in situ hybridization (FISH) and immunofluorescence microscopy

The RNA-FISH assay was performed as previously described.^81^ A 0.856 kb fragment of *LETN* was amplified using the primer set (TCAGCTCTCTGCTCAAGTA and ACATGTTAAGCATACTGCGG). The fragment was then cloned into pGEM-T (Promega). The *LETN* probe was synthesized with DIG RNA labeling Mix (Roche) and SP6 polymerase (Thermo), according to the manufacturers’ instructions.

The immunofluorescence assays were done as previously described.^81,86^ Coverslips in 24-well plates were pretreated with 50 µg/mL poly-D-lysine (Beyotime) before planting the cells. The cells were fixed with 4% formaldehyde solution and 1% acetic acid mixture for 10 min and then washed with 1× PBS solution for three times at room temperature (RT). Subsequently, the cells were permeabilized with 0.5% Triton X-100 in PBS solution for 5 min, rinsed, and then blocked with 3% BSA in TBST at RT for 1 h. The slides were then incubated with primary antibody diluted in blocking solution at 4 °C overnight. The slides were washed with TBST and then incubated with the secondary antibodies for Alexa Fluor^®^ 488(1:400, Donkey anti-Mouse, abcam), Alexa Fluor^®^ 594 (1:400, Donkey anti-sheep, abcam) at RT for 1 h. The coverslips were stained with 1 mg/mL DAPI (Sigma) for 5 min and washed three times for 5 min with TBST. Next, cells were mounted on a slide with Prolong Gold Antifade Mountant (Thermo) and stored in a dark chamber. The slides were imaged with an inverted Nikon N-SIM/A1 microscope which is a combination SIM and laser scanning confocal microscope.

Colocalization of the fluorescence signals for each cell was quantified by the method of Pearson’s correlation coefficients (PCC).^46^ The maximal value of PCC (1.0) indicates perfect colocalization between two fluorescence signals in a cell, whereas PCC = 0 indicates no colocalization. Specifically, the analysis was done with the NIS-Elements-AR software by following the software instruction. For each condition, about 20 cells were randomly selected, and their PCC values were reported.

### Time-lapse microscopy

HUH7 cells were cultured on 20 mm glass bottom cell dish and co-transfected with pLV-NPM1-mcherry, pMS2-GFP and pcDNA3.1-*LETN*-MS2 plasmids. After 24 h, cells were put under a Nikon A1R HD25 Confocal Microscope supplemented with live cell imaging environment control system. Cells were photographed every 15 min for up to 12.5 h. All of the time-lapse images were processed using Imaris ×64 9.3.1 software.

### FLIM-FRET assay

FLIM-FRET measures donor–receptor energy transfer and was performed as previously described.^87^ HUH7 cells were transfected with *LETN* siRNA or control siRNA in advance. Twenty-four hours later, cells were transfected with GFP-NPM1 and mCherry-NPM1 plasmids. After 48 h, the cells were carried out on FV1200 Confocal/FLIM/FCS (Olympus). In brief, GFP fluorescence was excited at 488 nm, emission decays were collected on the pixel-by-pixel basis in the epi-fluorescence mode. The proximity should be manifested by shortening of GFP fluorescence lifetime since excitation is then transferred from the donor eGFP to the acceptor mCherry. The lifetime images were generated by a robust ‘fast-FLIM’ approach. FLIM data were analyzed by the SymPhoTime64 software (PicoQuant). FRET is demonstrated by shortening of the eGFP lifetime in cells. In all experiments, about 30 cells were measured in each group.

### MS2bp-GFP RNA pull down

pcDNA3.1-MS2 or pcDNA3.1-*LETN*-MS2 was co-transfected with pMS2-GFP (Addgene) into HUH7 cells. After 48 h, cells (10 million) were harvested and lysed with 1 mL native lysis buffer (50 mM Tris, pH 7.4, 150 mM NaCl, 0.5% NP-40, 0.5 mM PMSF, 2 mM RVC, protease inhibitor cocktail (Roche)) followed by sonication. After centrifuging at 12,000 rpm for 30 min at 4 °C, the supernatant was pre-cleared with 10 μL Dynabeads Protein-G. The samples were then incubated with GFP antibody (3 µg per reaction; ab290, abcam) for 2 h at 4 °C, followed by addition of 20 μL Dynabeads Protein-G to the mixture and incubation overnight at 4 °C on a rotating shaker. Next, the samples were washed with wash buffer (50 mM Tris, pH 7.4, 300 mM NaCl, 0.5% NP-40, 0.5 mM PMSF, 2 mM RVC, protease inhibitor cocktail (Roche, 4693124001)) for four times at 4 °C. Finally, the magnetic beads were resuspended in 20 μL 1× SDS loading buffer and boiled for 10 min. The samples were analyzed by 10% SDS-PAGE and visualized by Fast Silver Stain Kit (P0017s, Beyotime) according to the manufacturer’s instructions. The proteins were recovered from the bands and subjected to mass spectrometry analysis.

### RIP assays

RIP was performed as previously described.^16^ For the native RIP assay, 10 million HUH7 cells were harvested and lysed with 600 μL RIP buffer (50 mM Tris, pH 7.4, 150 mM NaCl, 0.5% NP-40, 0.5 mM PMSF, 2 mM RVC, protease inhibitor cocktail (Roche, 4693124001)), followed by sonication (power 10 for four cycles, 10 s for sonication and 50 s for rest). After centrifuging at 12,000 rpm for 15 min, the supernatant was pre-cleared with 10 μL Dynabeads Protein G. The samples were then incubated with 3 µg of anti-NPM1 antibody (ab10530, abcam) for 2 h at 4 °C, and 20 μL Dynabeads Protein-G were added to the mixture and incubated overnight at 4 °C on a rotating shaker. Next, the antibody-bead slurry was washed five times at 4 °C with wash buffer (50 mM Tris, pH 7.4, 300 mM NaCl, 0.5% NP-40, 0.05% sodium deoxycholate, 0.5 mM PMSF, 2 mM RVC, protease inhibitor cocktail (Roche, 4693124001)). The RNA–protein complex was resuspended with proteinase K buffer (117 µL of wash buffer, 15 µL of 10% SDS, and 9 µL of proteinase K (20 mg/mL)). RNA was purified using TRIzol followed by RT-qPCR analysis.

For the formaldehyde crosslinking RIP assay, 1% formaldehyde was used to fix the cells for 10 min at RT. The crosslinking was quenched by the addition of glycine to a final concentration of 0.14 M, and the cells were incubated at RT for 30 min. After centrifuging at 1200 rpm for 5 min, the cell pellet was lysed with 600 μL RIP buffer, followed by sonication (power 10 for four cycles, 10 s for sonication and 50 s for rest). After centrifuging at 12,000 rpm for 15 min, the supernatant was pre-cleared with 10 μL Dynabeads Protein G. The sample were then incubated with 3 µg of anti-NPM1 antibody (ab10530, abcam) for 2 h at 4 °C, and 20 μL Dynabeads Protein-G were added to the mixture and incubated overnight at 4 °C on a rotating shaker. Next, the antibody-bead slurry was washed four times at 4 °C with wash buffer I (50 mM Tris, pH 7.4, 500 mM NaCl, 1% NP-40, 0.5% sodium deoxycholate, 0.5 mM PMSF, 2 mM RVC, protease inhibitor cocktail (Roche, 4693124001)), and wash buffer II (50 mM Tris, pH 7.4, 500 mM NaCl, 1% NP-40, 0.5% sodium deoxycholate, 1 M Urea, 0.5 mM PMSF, 2 mM RVC, protease inhibitor cocktail (Roche, 4693124001)), respectively. Reverse crosslinking was performed by adding 8 μL 5 M NaCl, followed by addition of 2 μL proteinase K (20 mg/mL) at 55 °C for 2 h. RNA was purified using TRIzol followed by RT-qPCR analysis.

### Biotin-labeled RNA pull-down assay

The *LETN* RNA truncations were labeled with biotin-16-UTP by in vitro transcription with SP6 polymerase and DIG RNA labeling Mix (11685597910, Roche) according to the manufacturers’ instructions. The biotin-labeled RNA was captured with Dynabeads MyOne Streptavidin C1 (65002, invitrogen) according to the recommended protocol. Five million HUH7 cells were harvested and lysed with lysis buffer (50 mM Tris, pH 7.4, 150 mM NaCl, 0.5% NP-40, 0.5 mM PMSF, 2 mM RVC, protease inhibitor cocktail (Roche)) followed by sonication (power 10 for four cycles, 10 s for sonication and 50 s for rest). This mixture was then added into the cell lysate, followed by incubation for 4 h at 4 °C. After washing for four times with wash buffer (50 mM Tris, pH 7.4, 300 mM NaCl, 0.5% NP-40, 0.5 mM PMSF, 2 mM RVC, protease inhibitor cocktail (Roche)) at 4 °C, 5 min each, the complex was boiled for 10 min at 100 °C in 1× SDS loading buffer and the protein pulled down was analyzed by western blotting.

### In vitro RNA–protein Interaction assay

The *LETN* RNA fragment was transcribed in vitro with SP6 polymerase and labeled with biotin-16-UTP. The *LETN* fragment was incubated with purified NPM1 or the mutant proteins in the binding buffer (50 mM Tris, pH 7.4, 150 mM NaCl, 0.5% NP-40, 0.5 mM PMSF, 2 mM RVC and protease inhibitor cocktail (Roche)) for 2 h at 4 °C. Meanwhile, Dynabeads Protein-G were incubated with 3 µg of anti-NPM1 antibody (ab52644, abcam) for 2 h at 4 °C. The sample in the binding buffer was added to the antibody-bead slurry and incubated overnight at 4 °C on a rotating platform. Next, the mixtures were washed with wash buffer (50 mM Tris, pH 7.4, 300 mM NaCl, 0.5% NP-40, 0.5 mM PMSF, 2 mM RVC, protease inhibitor cocktail (Roche)) for five times at 4 °C. An aliquot of the sample was transferred to a new microcentrifuge tube and kept as quality control via western blotting. The RNA-protein complex was resuspended with proteinase K buffer (117 µL of wash buffer, 15 µL of 10% SDS, and 9 µL of proteinase K (20 mg/mL)). RNA was purified using TRIzol followed by RT-qPCR analysis.

### Chromatin immunoprecipitation

HUH7 cells (10 million) were harvested and fixed with 1% formaldehyde in 10 mL PBS for 10 min at 37 °C. The crosslinking was quenched by the addition of glycine to a final concentration of 0.14 M, and the cells were incubated at RT for 30 min. The cell pellet was lysed with 600 μL lysis buffer (50 mM Tris, pH 7.4, 150 mM NaCl, 0.5% NP-40, 0.5 mM PMSF, 2 mM RVC, protease inhibitor cocktail (Roche)) at 4 °C for 30 min. The cell lysate was sonicated to achieve 300–700 bp DNA fragments. The lysate was cleared by centrifuging at 12,000× *g* for 10 min at 4 °C. An aliquot of the sheared chromatin was transferred to a new microcentrifuge tube and kept as a reference of input DNA. The remaining sample was mixed with Dynabeads at 4 °C for 2 h to eliminate non-specific binding. Meanwhile the beads were incubated for 2 h at 4 °C with 4 µg anti-NPM1 antibody or anti-IgG antibody and washed three times with PBS/BSA. Then the sample was added to antibody–bead slurry and incubated overnight at 4 °C on a rotating platform. Next, the slurry was washed five times with RIPA buffer (50 mM Hepes, pH 8.0, 1% NP-40, 0.7% DOC, 0.5 M LiCl, 1× protease inhibitor cocktail). The complex was eluted by adding 100 μL fresh-prepared elution buffer (10 mM Tris, pH 8.0, 1 mM EDTA, and 1% SDS) with rotation at RT for 15 min. Reverse crosslinking was performed at 65 °C overnight by adding 8 μL 5 M NaCl, followed by supplemented with 2 μL proteinase K (20 mg/mL) at 55 °C for 2 h. Genomic DNA was isolated using a Tiangen DNA purification kit and then incubated with 1 µL of RNase (20 mg/mL) at 37 °C for 1 h. Finally, the DNA was re-isolated using AMpure XP beads.

### Co-immunoprecipitation

The cells were harvested and lysed with Pierce IP lysis buffer (87787, Thermo) supplemented with 1 mM PMSF and protease inhibitor cocktail (Roche) followed by sonication (power 10 for four cycles, 10 s for sonication and 50 s for rest). One tenth of the lysate was taken out for a reference of input. Meanwhile the Dynabeads Protein-G were incubated for 2 h at 4 °C with 4 µg anti-NPM1 antibody or anti-IgG antibody and washed three times with PBS/BSA. The remaining lysate was then incubated with anti-NPM1-Dynabeads Protein-G slurry and incubated overnight at 4 °C on a rotating platform. Next, the beads were washed for four times with wash buffer (50 mM Tris, pH 7.4, 300 mM NaCl, 0.5% NP-40, 0.5 mM PMSF, 2 mM RVC, protease inhibitor cocktail (Roche)) at 4 °C. After discarding the supernatant, the beads were resuspended with 40 μL 1× SDS loading buffer and heated at 100 °C for 10 min, followed by western blot analysis.

### In vitro NPM1 oligomerization assay

Purified NPM1 in the binding buffer (50 mM Tris, pH 7.4, 150 mM NaCl, 0.5% NP-40, 0.5 mM PMSF) was subjected to sonication (ten cycles, 5 s for sonication and 15 s for rest). The 5′ fragment of *LETN* was synthesized by in vitro transcription, which is around 2000 nt. The protein–RNA binding was carried out at RT for 30 min, with indicated concentrations of the protein and the RNA in the binding buffer supplemented with 2 mM RVC and protease inhibitor cocktail (Roche). The products were immediately loaded into a 10% native PAGE gels without SDS followed by western blotting analysis.

### Transmission electron microscopy

Huh7 cells were fixed with 2.5% glutaraldehyde and 2.0% paraformaldehyde buffer with pH 7.2. The cells were then kept at 4 °C overnight. Next, the cells were washed with PB (0.1 M, pH 7.2) buffer for three times, and treated with 1.5% K_3_Fe(CN)_6_ + 1% OsO_4_ buffer and kept at 4 °C for 1 h. The cells were washed with PB (0.1 M, pH 7.2) buffer and ddH_2_O for three times (10 min each wash), and then treated with 1% uranyl acetate and kept at RT for 1 h. Cells were treated in turn by dehydrated in ethanol (30%, 50%, 70%, 90%, and 100%; 10 min each), 100% epoxy propane twice, PO and 812 resin (1:2; 2:1) for 30 min at RT, and resin alone overnight. The sample was embedded and made into ultrathin sections. Images were obtained with a H-7650B TEM at 80 kV.

### Neural differentiation from human ESCs

Human ES cell line H9 (WA09 line, NIH registry 0046) was maintained on Matrigel (Corning #354277)-coated dishes with the E8 medium (Thermo #A1517001) and detached with 500 μM EDTA (Thermo #15575020, diluted as 1:1000 with PBS before use) for passage. Neural induction was performed majorly according to the published method. Briefly, before neural induction, human ESCs were dissociated into single cells with TrypLE (Thermo #12605028) and plated in Matrigel-coated 12-well plate at the density of 70,000 cells/well in E8 medium supplied with 10 μM Y27632 (Selleck #S1049). Once the cells reached 95% confluence, initiate neural induction by changing the medium to neural induction medium containing DMEM/F12 (Sigma #D6421), Neurobasal medium (Thermo #21103049) at 1:1, 2 mM L-glutamine (Thermo #A2916801), 1× glutaMAX (Thermo #35050061), 0.5× NEAA (Thermo #11140050), 0.5× N2 (Thermo #17502048), 0.5× B27 (Thermo #17504044), 5 µg/mL insulin (Sigma #19278) and 100 μM 2-Mercaptoethanol (Thermo #21985023). 1 μM Dorsomorphin (Selleck #S7840) and 10 μM SB431542 (Selleck #S1067) were supplied into neural induction medium freshly. The human ESCs were cultured in this medium for 14 days and showed neural epithelial cell morphology. Then the cells were dissociated with Accutase (STEMCELL Tech. #07920) and split 1:3 in poly-l-ornithine (Sigma #4957) and Laminin (Sigma #L2020)-coated 12-well plate in neural induction medium supplied with 20 ng/mL bFGF (Thermo #PHG0021L) for another 8 days. After that, the induced NPCs were dissociated with Accutase and split 1:3 and cultured in poly-l-ornithine and Laminin-coated 12-well plate in neural progenitor medium containing 1 mM sodium pyruvate (Thermo #11360070), 2 mM L-glutamine, 1 mM N-acetyl-cysteine (Sigma #A9165), 1× N2, 1× B27, 10 ng/mL bFGF and 10 ng/mL EGF.

### Lentiviral infection and immunofluorescence of induced NPCs

Two days after plating, the induced NPCs were affected with lentivirus carrying mock sequence and coding sequence of shRNA against *LETN*. Four days after infection, the NPC cultures were fixed with 4% polyformaldehyde. Then the cells were blocked with blocking buffer (5% BSA, Triton X-100 in PBS), and incubated with anti-Ki67 antibody (Thermo #PA5-19462) 1:500 and Alexa Fluor 488-conjugated antibodies (Thermo #R37116). The nucleus was counterstained with DAPI.

### Overall survival analysis

After allocating the patients into different groups, Kaplan–Meier survival curves were prepared for each of the patient groups. The clinical data were downloaded from the TCGA data portal. The R package ‘survival’ was used for comparison of the patient survival rates among different subgroups. The statistical significance of the prognosis difference was inferred with the log-rank test.

### Statistical analysis

Pair-wise comparisons of the experimental data were done with GraphPad Prism 6 or Excel, and the statistical analysis was performed with Student’s *t*-test unless indicated as other methods. Unless specified on the figure, all the statistical analyses were done between the experimental condition and the control (marked as siNC, shNC, EV, sgEV, or ASO-NC for the gene perturbation studies, IgG or anti-GFP for the RIP assays). The significance levels were marked by NS (not significant), *(*P*-value < 0.05), **(*P*-value < 0.01), and ***(*P*-value < 0.001). The error bars on the figures represent standard deviation (SD) of at least three biological replicates.

## Supplementary information

Supplementary information, Figure S1

Supplementary information, Figure S2

Supplementary information, Figure S3

Supplementary information, Figure S4

Supplementary information, Figure S5

Supplementary information, Figure S6

Supplementary information, Figure S7

Supplementary information, Figure S8

Supplementary information, Figure S9

Supplementary information, Figure S10

Supplementary information, Figure S11

Supplementary information, Figure S12

Supplementary information, Figure S13

Supplementary information, Figure S14

Supplementary information, Figure S15

Supplementary information, Figure S16

Supplementary information, Figure S17

Supplementary information, Figure S18

Supplementary information, Figure S19

Supplementary information, Figure S20

Supplementary information, Figure S21

Supplementary information, Figure S22

Supplementary information, Figure S23

Supplementary information, Figure S24

Supplementary information, Figure S25

Supplementary information, Figure S26

Supplementary information, Video S1

Supplementary information, Video S2

Supplementary information, Video S3

Supplementary information, Video S4

Supplementary information, Video S5

Supplementary information, Video S6

Supplementary information, Video S7

Supplementary information, Video S8

Supplementary information, Video S9

Supplementary Video Legend

## Data Availability

The gene expression datasets generated in this study are available in the GEO database repository (GEO accession ID: GSE140708).
