# Supplementary material for: Mutual dependency between lncRNA LETN and protein NPM1 in controlling the nucleolar structure and functions sustaining cell proliferation
Source: Cell Res. 2021 Jan 11;31(6):664–83. doi: 10.1038/s41422-020-00458-6 (PMC8169757; doi:10.1038/s41422-020-00458-6)
Supplement: Supplementary file 1 — Supplementary information, Figure S1 [file 41422_2020_458_MOESM1_ESM.pdf]

Figure S1

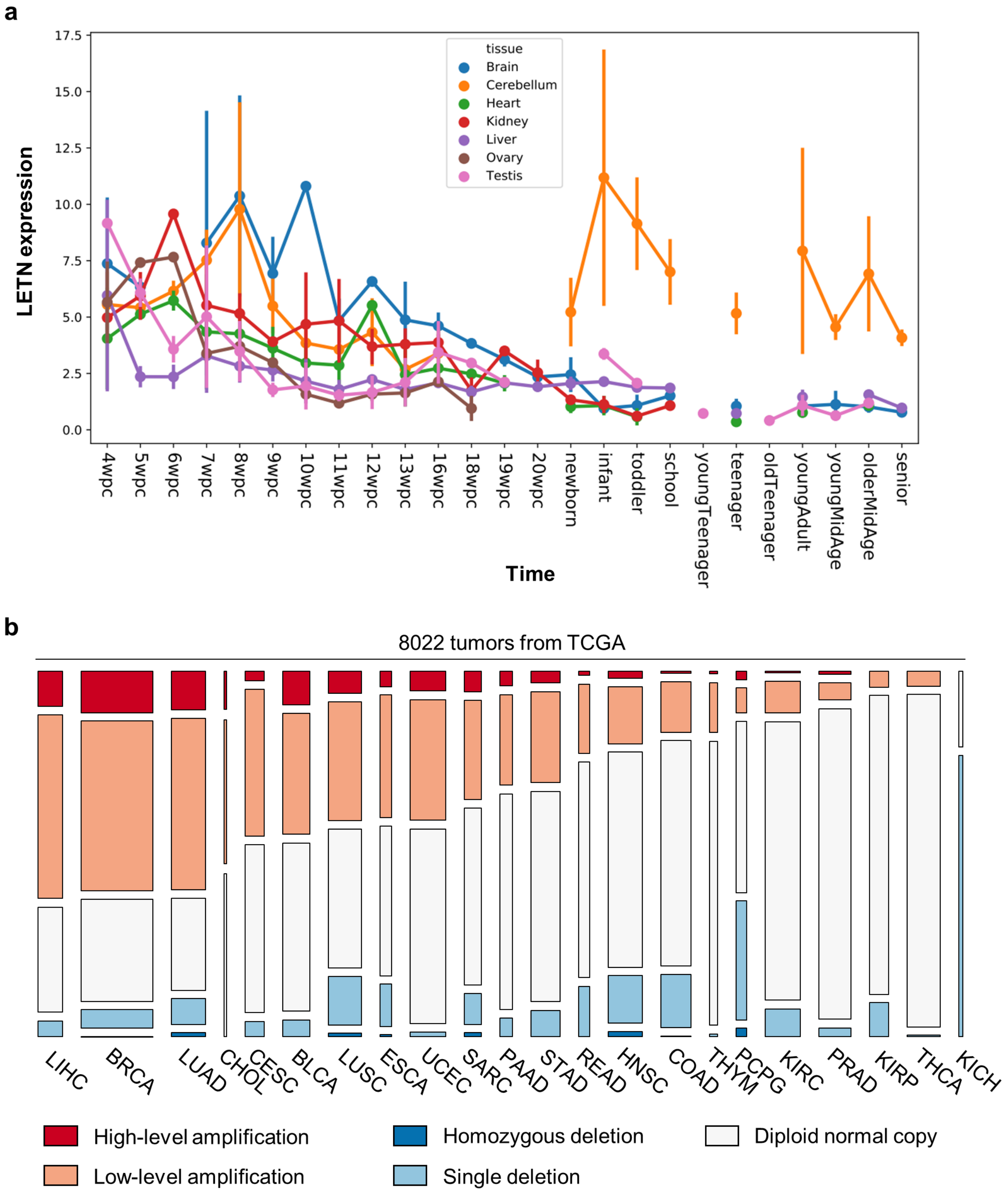

Fig. S1: Expression levels of LETN in the human organs and the landscapes of LETN CNVs in tumors from 22 cancers.

a The LETN expression levels in the main organs at different ages before and after birth. The data was obtained from the study of Sarropoulos, I., et al., Nature, 2019, which provided expression

dynamics of lncRNAs at differential developmental stages across human organs.

b DNA copy number variations of LETN in the tumors of different cancer types. The datasets were obtained from TCGA. The width of each column is proportional to the cohort size of each cancer.
