# Supplementary material for: Mutual dependency between lncRNA LETN and protein NPM1 in controlling the nucleolar structure and functions sustaining cell proliferation
Source: Cell Res. 2021 Jan 11;31(6):664–83. doi: 10.1038/s41422-020-00458-6 (PMC8169757; doi:10.1038/s41422-020-00458-6)
Supplement: Supplementary file 2 — Supplementary information, Figure S2 [file 41422_2020_458_MOESM2_ESM.pdf]

Figure S2

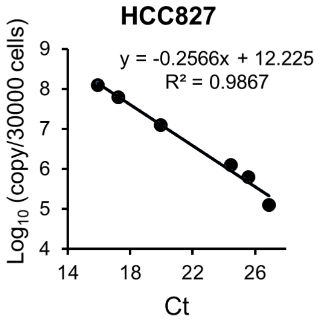

	Ct	copy/cell
LETN	21.284	193.3883
HOTTIP	27.008	6.569097
NEAT1	18.957	764.5556
SLERT	21.933	131.763
HOTAIR	32.045	0.335039
ACTIN	13.28	21889.24

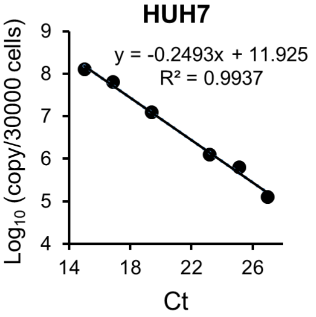

	Ct	copy/cell
LETN	20.859	176.854
HOTTIP	25.749	10.6845
NEAT1	16.882	1734.654
SLERT	22.136	84.974
HOTAIR	24.342	23.963
ACTIN	13.749	10478.89

Fig. S2: Estimations of RNA molecule numbers per cell.

For 2 cell lines, the average RNA copy numbers of LETN (and other transcripts as references) per cell were estimated by qPCR. Standard RNA spike-in was added into the cell lysates before the RT-qPCR assay. Left: standard curve generated by using different concentrations of the RNA spike-in. Y-axis: copies (log10) of the RNA Spike-in added into the lysate from 30000 cells; X-axis: the corresponding Ct values from the qPCR assay. Right: copies of the RNA molecules estimated by fitting the Ct values onto the standard curve.
