# Supplementary material for: Mutual dependency between lncRNA LETN and protein NPM1 in controlling the nucleolar structure and functions sustaining cell proliferation
Source: Cell Res. 2021 Jan 11;31(6):664–83. doi: 10.1038/s41422-020-00458-6 (PMC8169757; doi:10.1038/s41422-020-00458-6)
Supplement: Supplementary file 3 — Supplementary information, Figure S3 [file 41422_2020_458_MOESM3_ESM.pdf]

Figure S3

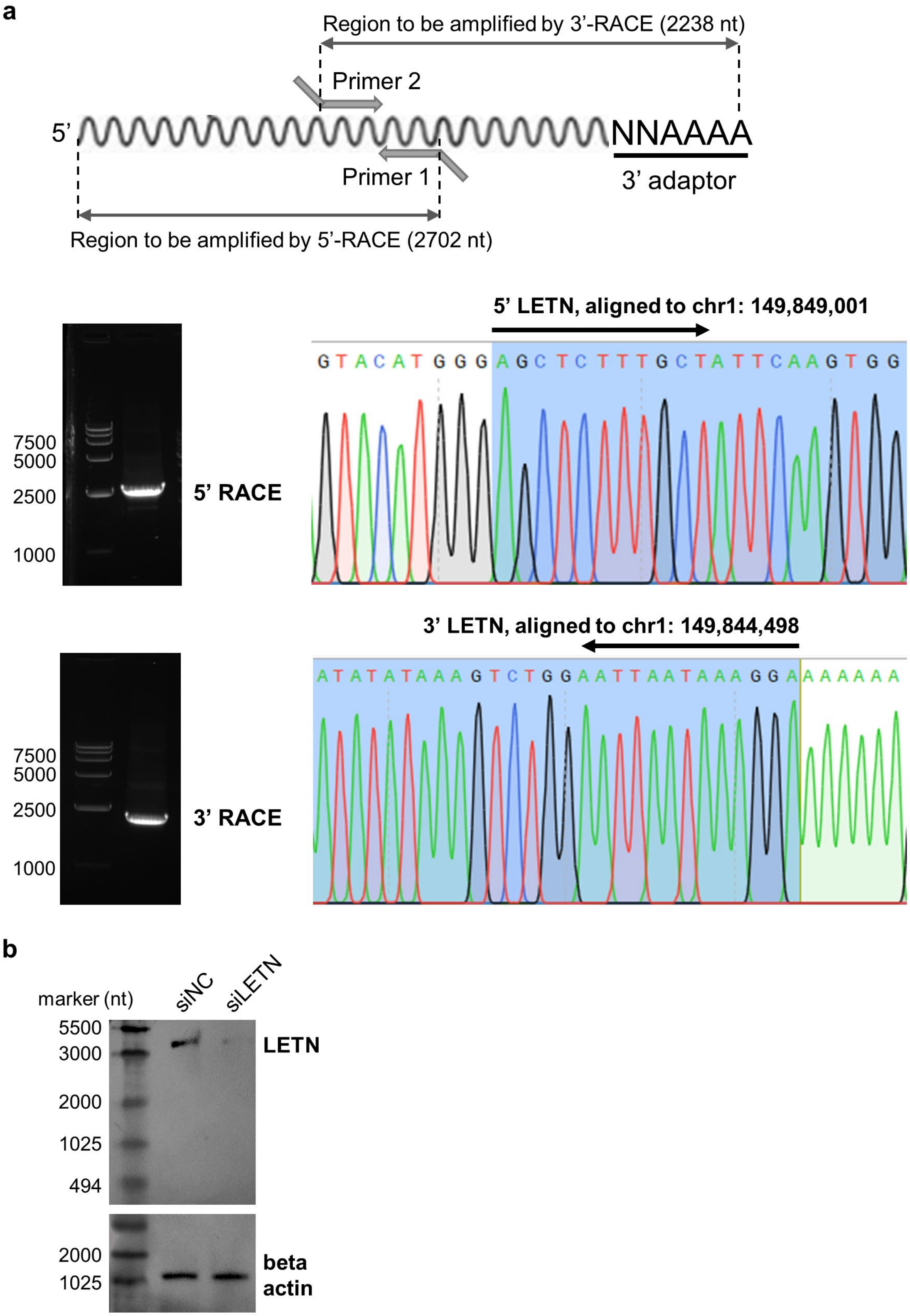
a
Region to be amplified by 3'-RACE (2238 nt)
Primer 2
5'
NNAAAA
Primer 1
3' adaptor
Region to be amplified by 5'-RACE (2702 nt)
5' LETN, aligned to chr1: 149,849,001
GTACATGGGAGCTCTTTGCTATTCAAGTGG
7500
5000
2500
1000
5' RACE
3' LETN, aligned to chr1: 149,844,498
ATATATAAAGTCTGGAATTAATAAAGGAAAAAAA
7500
5000
2500
1000
3' RACE
b
marker (nt)
siNC
siLETN
5500
3000
2000
1025
494
LETN
2000
1025
beta actin

Fig. S3: Confirmation of the LETN annotations on the genome.

a Top: Schematic drawing of the 5’- and 3’-RACE assays. Bottom: Agarose gel electrophoresis (left) and Sanger sequencing results (right) of the PCR products from the 5'-RACE and 3'-RACE with HUH7 cells.

b Northern blots with the probes for LETN and beta-actin mRNA under the conditions of control and siRNA-mediated LETN knockdown in HUH7 cells. Total RNA from the same number of cells under different conditions were used as inputs. Beta-actin mRNA was used as a loading control.
