# Supplementary material for: Mutual dependency between lncRNA LETN and protein NPM1 in controlling the nucleolar structure and functions sustaining cell proliferation
Source: Cell Res. 2021 Jan 11;31(6):664–83. doi: 10.1038/s41422-020-00458-6 (PMC8169757; doi:10.1038/s41422-020-00458-6)
Supplement: Supplementary file 4 — Supplementary information, Figure S4 [file 41422_2020_458_MOESM4_ESM.pdf]

Figure S4

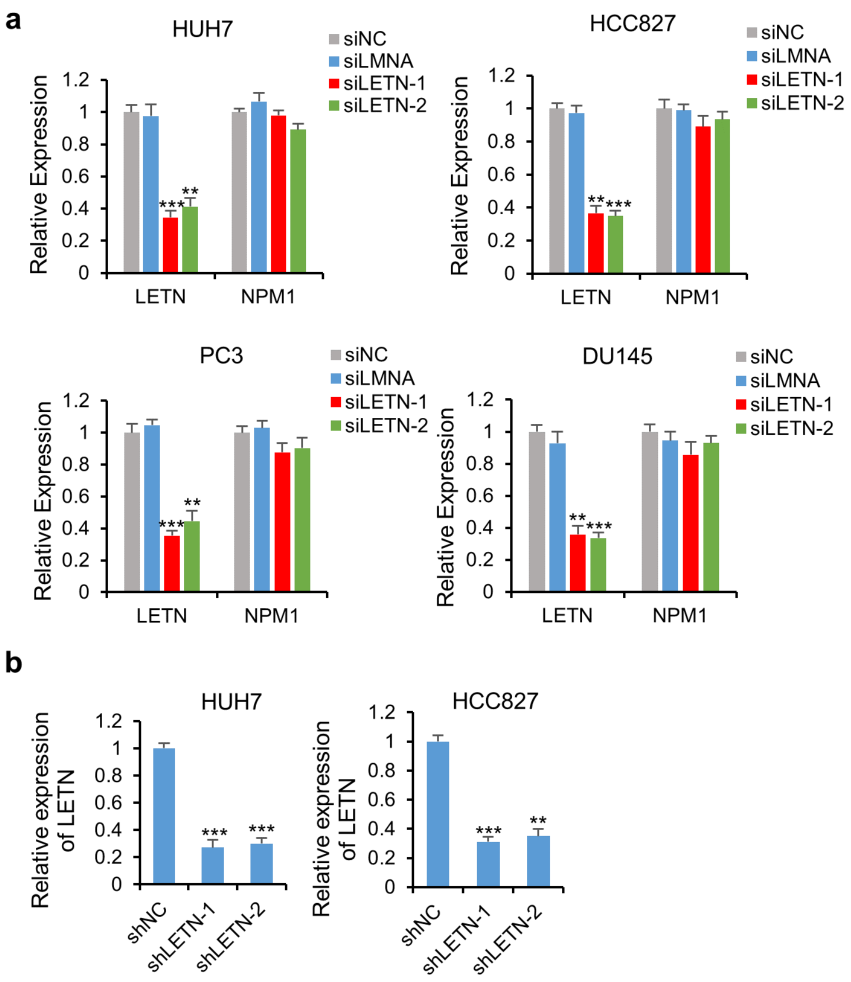

Fig. S4: Relative expression levels of LETN upon different types of perturbations in various cancer cell lines.

a Relative expression levels of LETN, measured by qPCR, in HUH7, HCC827, DU145 and PC3 cells after siRNA-mediated knockdown. The error bars represent the ± SD of 3 biological replicates.

b Relative expression levels of LETN, measured by qPCR, in HUH7 and HCC827 cells with lentivirus-mediated long-term knockdown of LETN with shRNA. The error bars represent the ± SD of 3 biological replicates.
