# Supplementary material for: Mutual dependency between lncRNA LETN and protein NPM1 in controlling the nucleolar structure and functions sustaining cell proliferation
Source: Cell Res. 2021 Jan 11;31(6):664–83. doi: 10.1038/s41422-020-00458-6 (PMC8169757; doi:10.1038/s41422-020-00458-6)
Supplement: Supplementary file 5 — Supplementary information, Figure S5 [file 41422_2020_458_MOESM5_ESM.pdf]

Figure S5

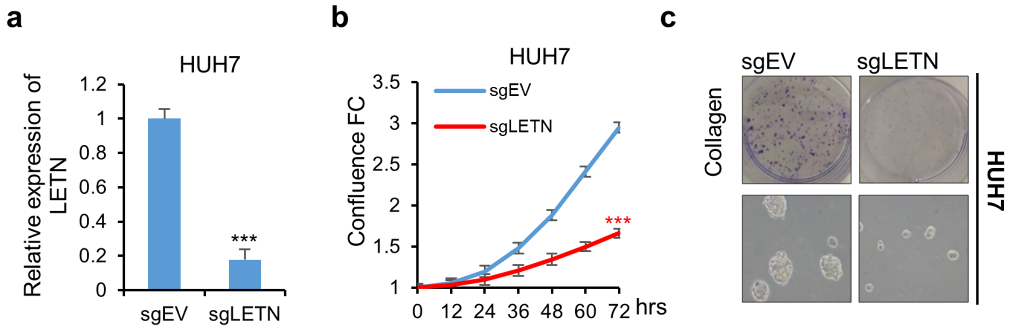

Fig. S5: Cell proliferation and colony formation upon knockout of LETN in HUH7 cells.

a Relative expression level of LETN, measured by qPCR, in HUH7 cell upon CRISPR-mediated knockout for LETN. The error bars represent the ± SD of 3 biological replicates.

b Cell proliferation curves of HUH7 upon CRISPR-Cas9 mediated LETN knockout. The error bars represent the ± SD of 3 biological replicates.

c Anchorage-dependent (top) or -independent (bottom) colony formation of HUH7 upon CRISPR-Cas9 mediated LETN knockout.
