# Supplementary material for: Mutual dependency between lncRNA LETN and protein NPM1 in controlling the nucleolar structure and functions sustaining cell proliferation
Source: Cell Res. 2021 Jan 11;31(6):664–83. doi: 10.1038/s41422-020-00458-6 (PMC8169757; doi:10.1038/s41422-020-00458-6)
Supplement: Supplementary file 7 — Supplementary information, Figure S7 [file 41422_2020_458_MOESM7_ESM.pdf]

Figure S7

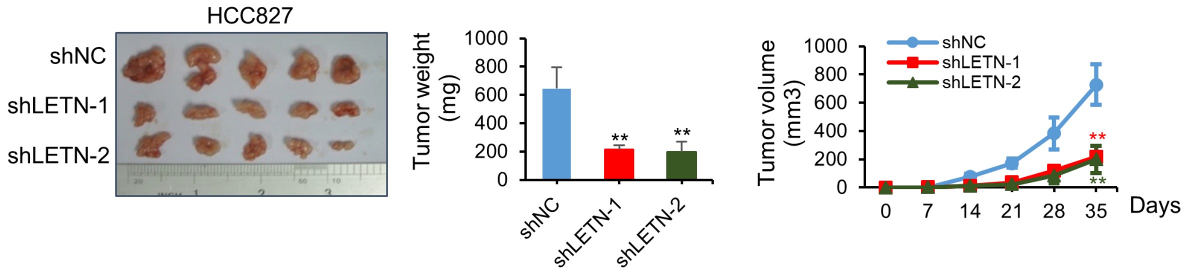

Fig. S7: Effects of LETN knockdown on tumor growth.

Images, weights, and growth records of the xenograft tumors in nude mice developed from the HCC827 cells (n=5) with lentivirus-mediated stable gene knockdown. The error bars represent the ± SD of the 5 tumors.
