# Supplementary material for: Mutual dependency between lncRNA LETN and protein NPM1 in controlling the nucleolar structure and functions sustaining cell proliferation
Source: Cell Res. 2021 Jan 11;31(6):664–83. doi: 10.1038/s41422-020-00458-6 (PMC8169757; doi:10.1038/s41422-020-00458-6)
Supplement: Supplementary file 8 — Supplementary information, Figure S8 [file 41422_2020_458_MOESM8_ESM.pdf]

Figure S8

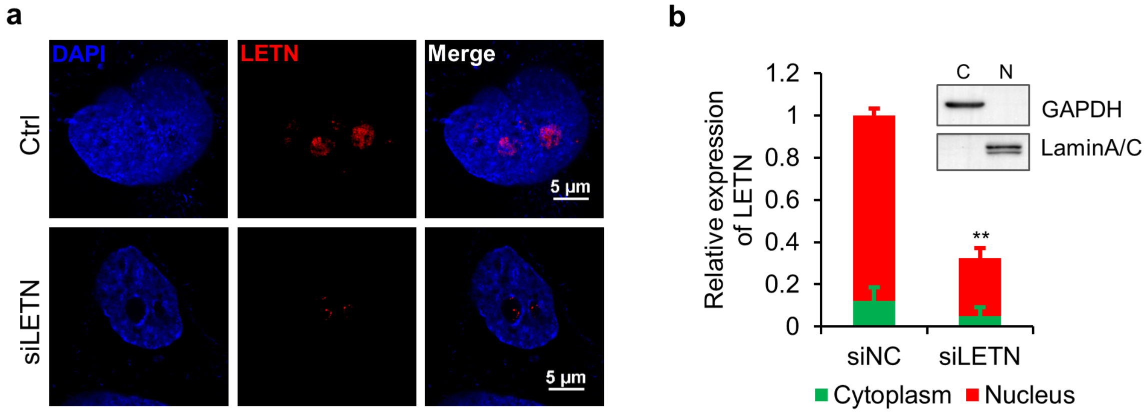

Fig. S8: Subcellular localization of LETN in HUH7 cells.

a Nucleus staining with 4',6-diamidino-2-phenylindole (DAPI) (blue) and RNA FISH assay of LETN (red) in HUH7 cells under the conditions of control and LETN knockdown.

b RT-qPCR of LETN in the cytosolic and nuclear fractions of HUH7 cells under the conditions of control and LETN knockdown. GAPDH and LaminA/C were probed with western blots to illustrate clean separation of the cytosolic and nuclear fractions.
