# Supplementary material for: Mutual dependency between lncRNA LETN and protein NPM1 in controlling the nucleolar structure and functions sustaining cell proliferation
Source: Cell Res. 2021 Jan 11;31(6):664–83. doi: 10.1038/s41422-020-00458-6 (PMC8169757; doi:10.1038/s41422-020-00458-6)
Supplement: Supplementary file 9 — Supplementary information, Figure S9 [file 41422_2020_458_MOESM9_ESM.pdf]

Figure S9

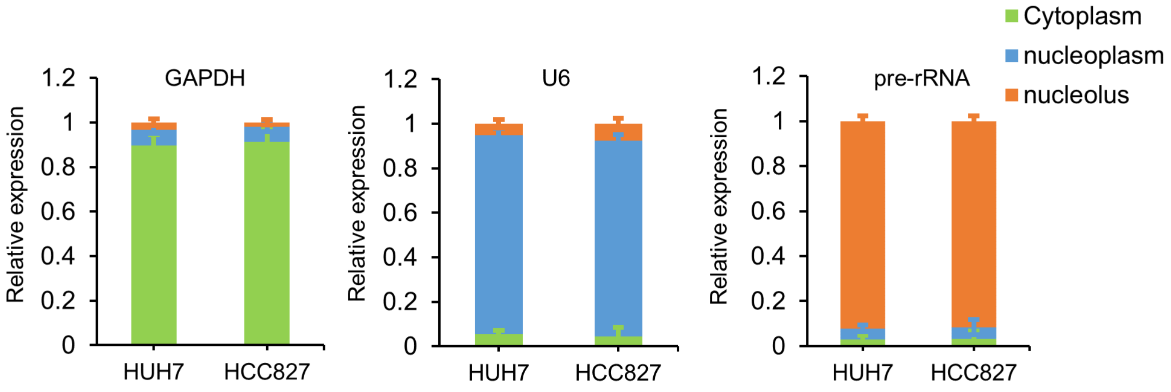

Fig. S9: Relative expression patterns of the RNA markers in the cytoplasm, nucleoplasm and nucleolus.

Supplementary to Fig. 2a. RT-qPCR of GAPDH, U6 and pre-rRNA in the cytoplasmic, nucleoplasmic and nucleolar fractions of HUH7 and HCC827 cells.
