# Supplementary material for: Mutual dependency between lncRNA LETN and protein NPM1 in controlling the nucleolar structure and functions sustaining cell proliferation
Source: Cell Res. 2021 Jan 11;31(6):664–83. doi: 10.1038/s41422-020-00458-6 (PMC8169757; doi:10.1038/s41422-020-00458-6)
Supplement: Supplementary file 10 — Supplementary information, Figure S10 [file 41422_2020_458_MOESM10_ESM.pdf]

**Figure S10**

a

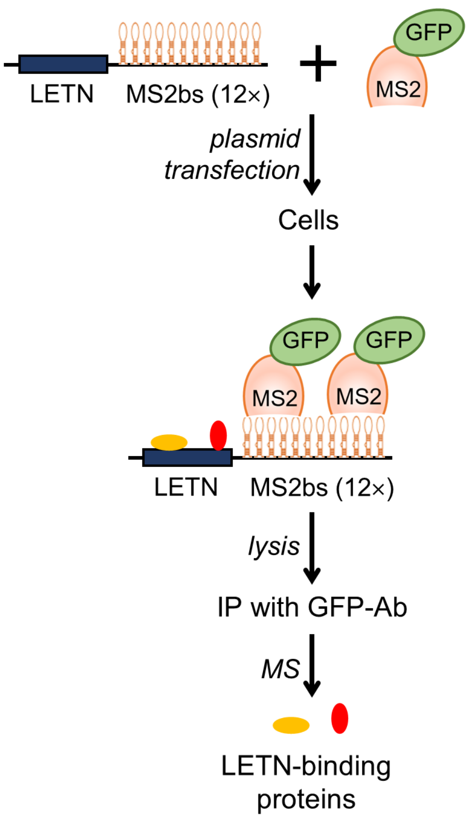


b

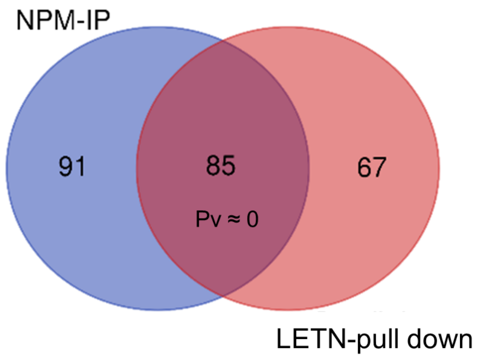


| | | | |
|---|---|---|---|
| NPM1 | TUBB4B | HEL-S-39 | RPL17 |
| HIST1H2AH | RAN | YWHAB | RPL10 |
| HIST1H2BJ | YWHAZ | RPS4X | RPL32 |
| HIST1H1C | SNRPD2 | RPL8 | MYL6 |
| UBC | RPL7 | RPL27 | RPL26 |
| H2AFZ | HMGA | DDX5 | ILF3 |
| HIST2H3PS2 | RPL6 | RPL24 | HSP90AB1 |
| HNRNPC | RPL13 | PCBP1 | RPS9 |
| HIST2H2AB | RPL11 | RPL34 | HSP90AA1 |
| H2AFY | RPL19 | SPTAN1 | RPL3 |
| PTBP1 | HNRNPAB | RPL35 | RPS25 |
| NCL | RPL4 | RPS26 | DDX39B |
| ILF2 | RPL21 | CIP29 | RPS16 |
| EEF1A1 | RPS3 | RPL29 | RPS13 |
| RSL1D1 | HNRNPA1 | RPS11 | RPS23 |
| RPL18 | HNRNPH1 | YBX1 | RPL9 |
| HNRNPK | CBX3 | RPS14 | RPL28 |
| RPL23A | RPS18 | RPS7 | RPS5 |
| ELAVL1 | RPS6 | SNRPA1 | H1FX |
| SFPQ | RPS8 | CFL1 | RPL7A |
| HNRPU | RPS2 | RPL31 | RPS19 |
| ENO1 | RPL12 | EIF6 | |

**Fig. S10: Proteins pulled-down by LETN-MS2-RIP or NPM1 immunoprecipitation.**

**a** Schematic description of the LETN-MS2-RIP assay. See Methods for details of the experiment.
**b** Overlap between the two groups of proteins pulled-out by the MS2-tagged LETN or NPM1. The overlapping proteins are provided in the list.
