# Supplementary material for: Mutual dependency between lncRNA LETN and protein NPM1 in controlling the nucleolar structure and functions sustaining cell proliferation
Source: Cell Res. 2021 Jan 11;31(6):664–83. doi: 10.1038/s41422-020-00458-6 (PMC8169757; doi:10.1038/s41422-020-00458-6)
Supplement: Supplementary file 11 — Supplementary information, Figure S11 [file 41422_2020_458_MOESM11_ESM.pdf]

Figure S11

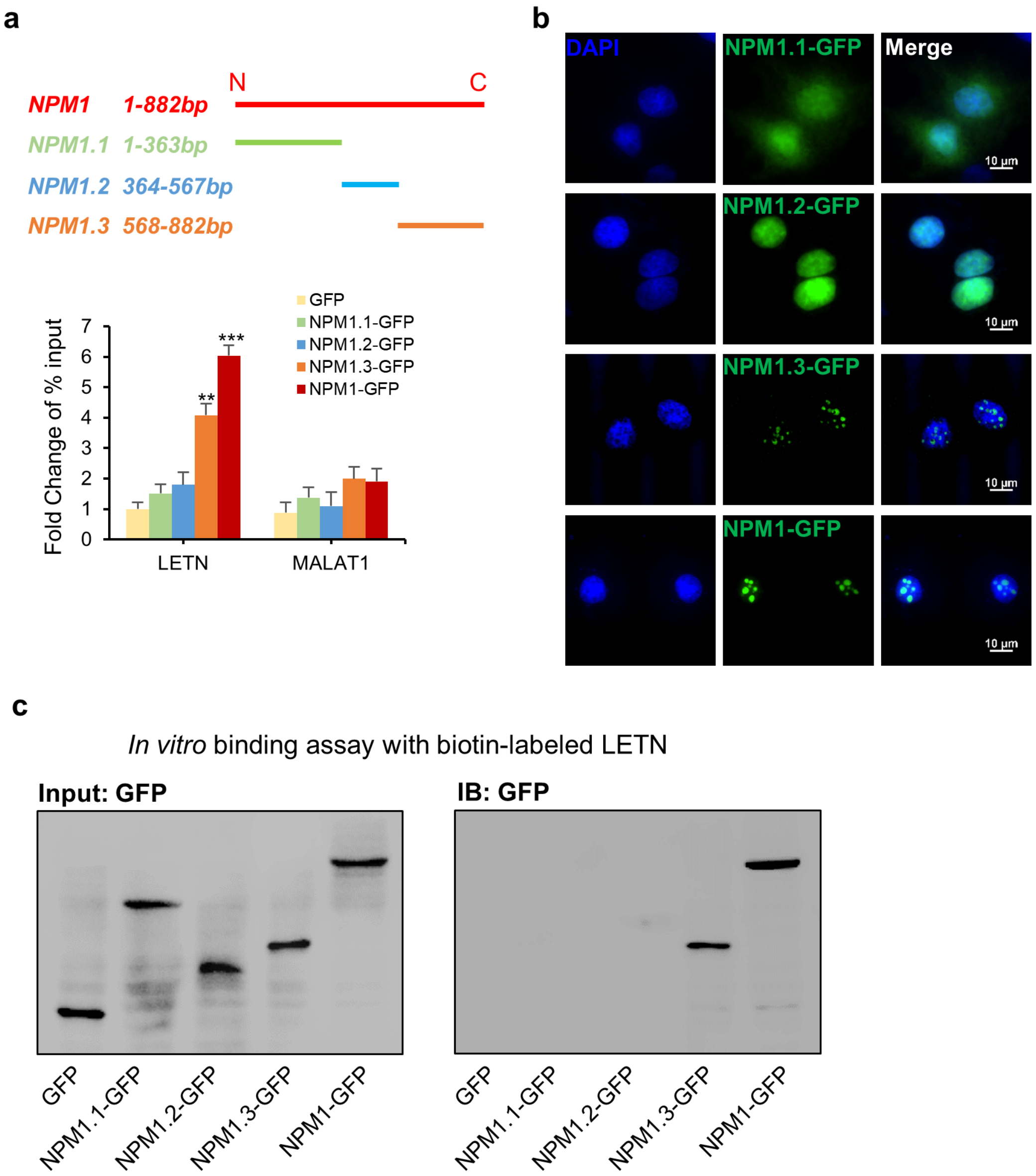

Fig. S11: Binding between LETN and the truncated NPM1 proteins.

a Truncated forms of NPM1 were fused with GFP and expressed in HUH7 cells. qPCR assays for LETN and MALAT1 (negative control) were performed after GFP-RIP.

b Nucleus staining with 4',6-diamidino-2-phenylindole (DAPI) (blue) and different truncated forms of NPM1 protein (green) in HUH7 cells.

c The truncated NPM1 proteins fused with GFP were expressed in NPM1-/- HUH7 cells, which were lysed and incubated with biotin-labeled LETN from *in vitro* transcription. GFP was then probed after enrichment of biotin from the lysate.
