# Supplementary material for: Mutual dependency between lncRNA LETN and protein NPM1 in controlling the nucleolar structure and functions sustaining cell proliferation
Source: Cell Res. 2021 Jan 11;31(6):664–83. doi: 10.1038/s41422-020-00458-6 (PMC8169757; doi:10.1038/s41422-020-00458-6)
Supplement: Supplementary file 12 — Supplementary information, Figure S12 [file 41422_2020_458_MOESM12_ESM.pdf]

Figure S12

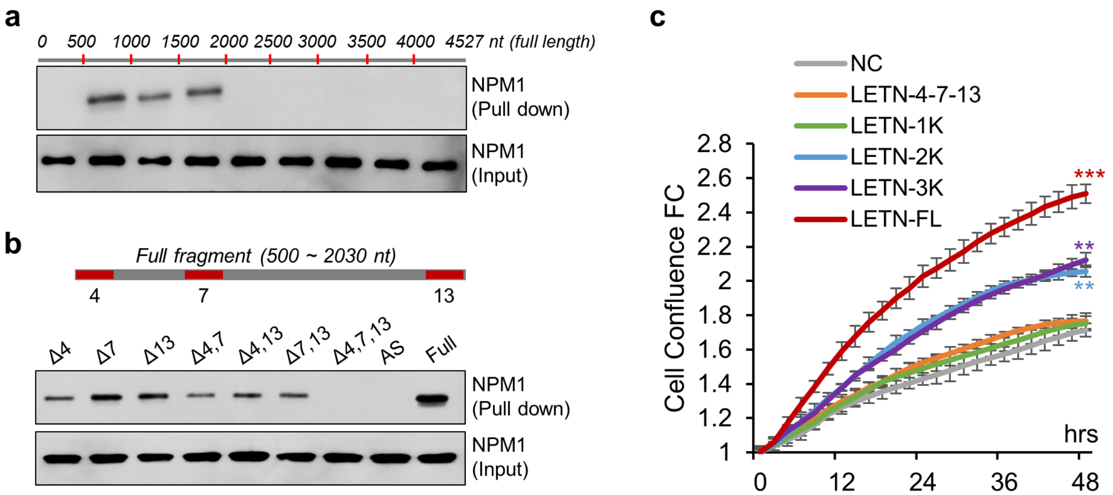

Fig. S12: Identifications of the NPM1-binding regions on LETN.

a, **b** Supplementary to Fig. 2d. Interactions between LETN fragments and NPM1. Biotin-labeled LETN fragments were used for protein pull-down in HUH7 cells, followed by western blots of NPM1. (**a**) 9 fragments (500 nt each) covered the full length of LETN. (**b**) The truncated LETN RNA (500-2030 nt) that lacks one, two, or all three of the fragments 4, 7, and 13 was used for protein pull-down.

c Rescue of HUH7 cell proliferation after knockdown of LETN, with different fragments of LETN. The error bars represent the ± SD of 3 biological replicates.
