# Supplementary material for: Mutual dependency between lncRNA LETN and protein NPM1 in controlling the nucleolar structure and functions sustaining cell proliferation
Source: Cell Res. 2021 Jan 11;31(6):664–83. doi: 10.1038/s41422-020-00458-6 (PMC8169757; doi:10.1038/s41422-020-00458-6)
Supplement: Supplementary file 13 — Supplementary information, Figure S13 [file 41422_2020_458_MOESM13_ESM.pdf]

Figure S13

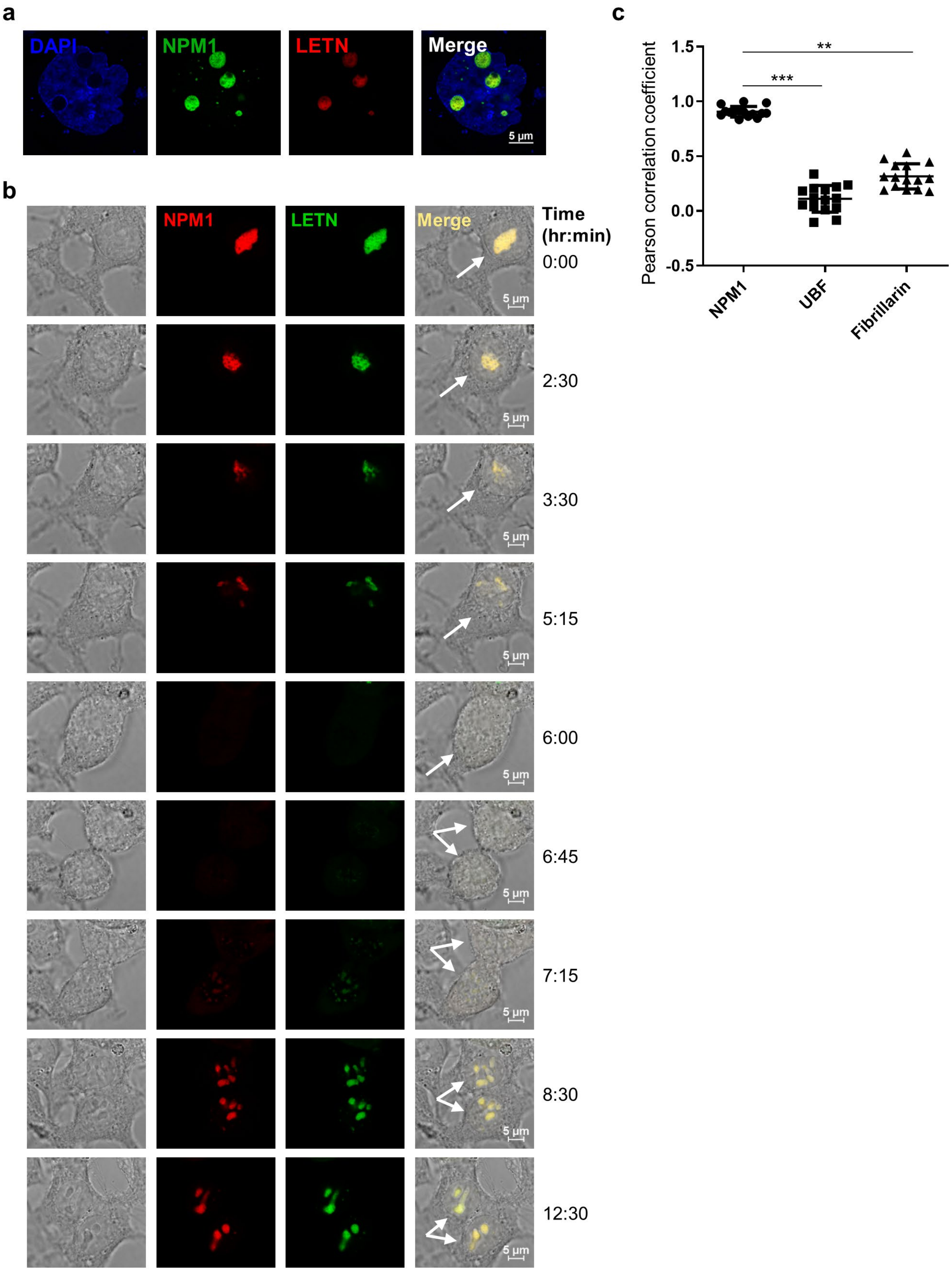
a
DAPI
NPM1
LETN
Merge
5 μm
b
NPM1
LETN
Merge
Time (hr:min)
0:00
2:30
3:30
5:15
6:00
6:45
7:15
8:30
12:30
5 μm
c
Pearson correlation coefficient
1.5
1.0
0.5
0.0
-0.5
**
***
NPM1
UBF
Fibrillarin

**Fig. S13: Colocalization between NPM1 and LETN.**

**a** Immunofluorescence staining of NPM1 (green), nucleus staining with DAPI (blue), and RNA FISH of LETN (red) in HUH7 cells.

**b** Supplementary to Fig. 2e. More representative time-lapse microscopy images showing dissociation and coaggregation of LETN and NPM1 in a dividing HUH7 cell. NPM1 was fused with mCherry (red) and MS2-tagged LETN was marked by MS2-GFP fusion protein (green). Refer to the Supplementary information, Video S1 for the full data.

**c** Supplementary to Fig. 2f, h. The degrees of colocalization between LETN and the three nucleolar marker proteins NPM1, UBF, and Fibrillarin were quantified by the Pearson's correlation coefficients (PCC). The maximal value of PCC (1.0) indicates perfect colocalization between two fluorescence signals in a cell, whereas PCC=0 indicates no colocalization. See Methods for the procedure of calculating the PCC values.
