# Supplementary material for: Mutual dependency between lncRNA LETN and protein NPM1 in controlling the nucleolar structure and functions sustaining cell proliferation
Source: Cell Res. 2021 Jan 11;31(6):664–83. doi: 10.1038/s41422-020-00458-6 (PMC8169757; doi:10.1038/s41422-020-00458-6)
Supplement: Supplementary file 14 — Supplementary information, Figure S14 [file 41422_2020_458_MOESM14_ESM.pdf]

Figure S14

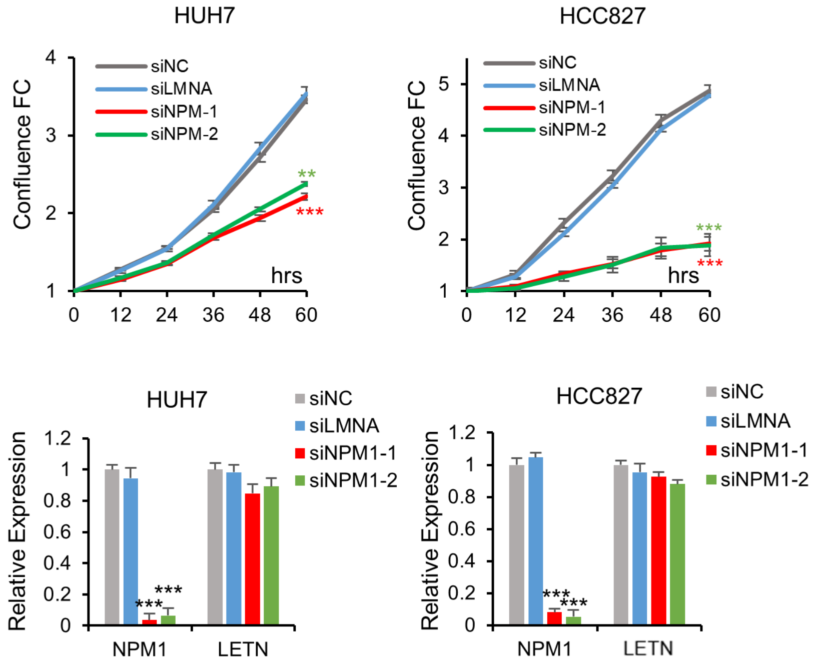

Fig. S14: Effect of NPM1 knockdown on cell proliferation.

Upper, cell proliferation curves of HUH7 and HCC827 cells upon siRNA-mediated knockdown of NPM1. Bottom, knockdown efficiencies of NPM1 in HUH7 and HCC827 cells. The error bars represent the ± SD of 3 biological replicates.
