# Supplementary material for: Mutual dependency between lncRNA LETN and protein NPM1 in controlling the nucleolar structure and functions sustaining cell proliferation
Source: Cell Res. 2021 Jan 11;31(6):664–83. doi: 10.1038/s41422-020-00458-6 (PMC8169757; doi:10.1038/s41422-020-00458-6)
Supplement: Supplementary file 15 — Supplementary information, Figure S15 [file 41422_2020_458_MOESM15_ESM.pdf]

Figure S15

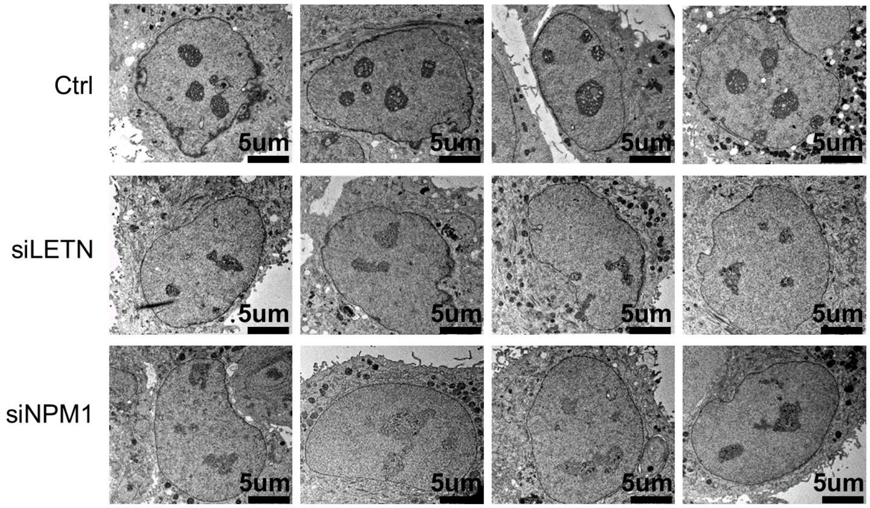

Fig. S15: Effects of NPM1 and LETN knockdown on the nucleolar morphology.

Supplementary to Fig. 3a. More examples of the nucleoli (obtained with TEM at 80kV, 7000 ×) of HUH7 cells under the conditions of control and LETN or NPM1 knockdown.
