# Supplementary material for: Mutual dependency between lncRNA LETN and protein NPM1 in controlling the nucleolar structure and functions sustaining cell proliferation
Source: Cell Res. 2021 Jan 11;31(6):664–83. doi: 10.1038/s41422-020-00458-6 (PMC8169757; doi:10.1038/s41422-020-00458-6)
Supplement: Supplementary file 16 — Supplementary information, Figure S16 [file 41422_2020_458_MOESM16_ESM.pdf]

Figure S16

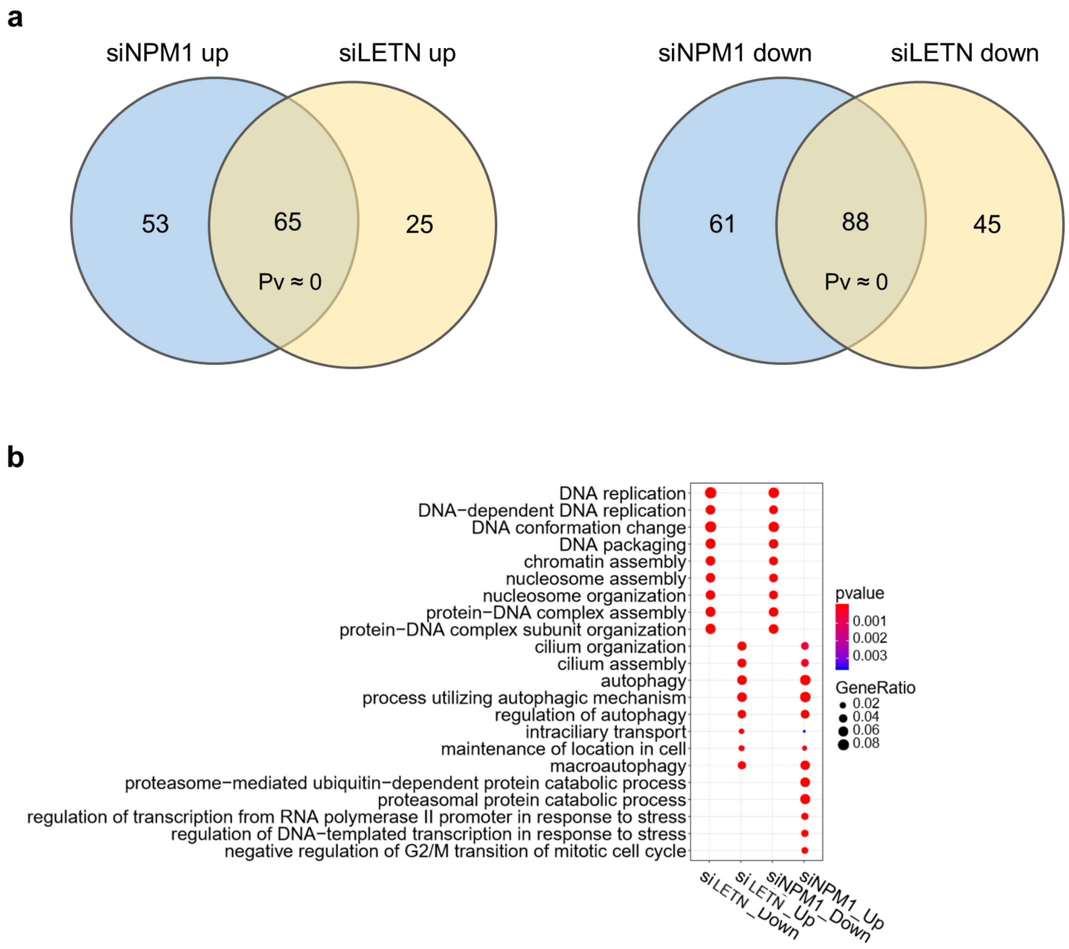

Fig. S16: Overlaps between the differentially expressed genes upon NPM1 and LETN knockdown.

a Supplementary to Fig. 3b. Overlaps between the differentially expressed genes upon LETN or NPM1 knockdown in HUH7 cells. P-values for both of the overlaps, by Fisher's Exact test, were close to 0.

b Enrichments of GO and KEGG functional annotations in the gene sets that were up- or down-regulated upon LETN or NPM1 knockdown in HUH7 cells.
