# Supplementary material for: Mutual dependency between lncRNA LETN and protein NPM1 in controlling the nucleolar structure and functions sustaining cell proliferation
Source: Cell Res. 2021 Jan 11;31(6):664–83. doi: 10.1038/s41422-020-00458-6 (PMC8169757; doi:10.1038/s41422-020-00458-6)
Supplement: Supplementary file 17 — Supplementary information, Figure S17 [file 41422_2020_458_MOESM17_ESM.pdf]

Figure S17

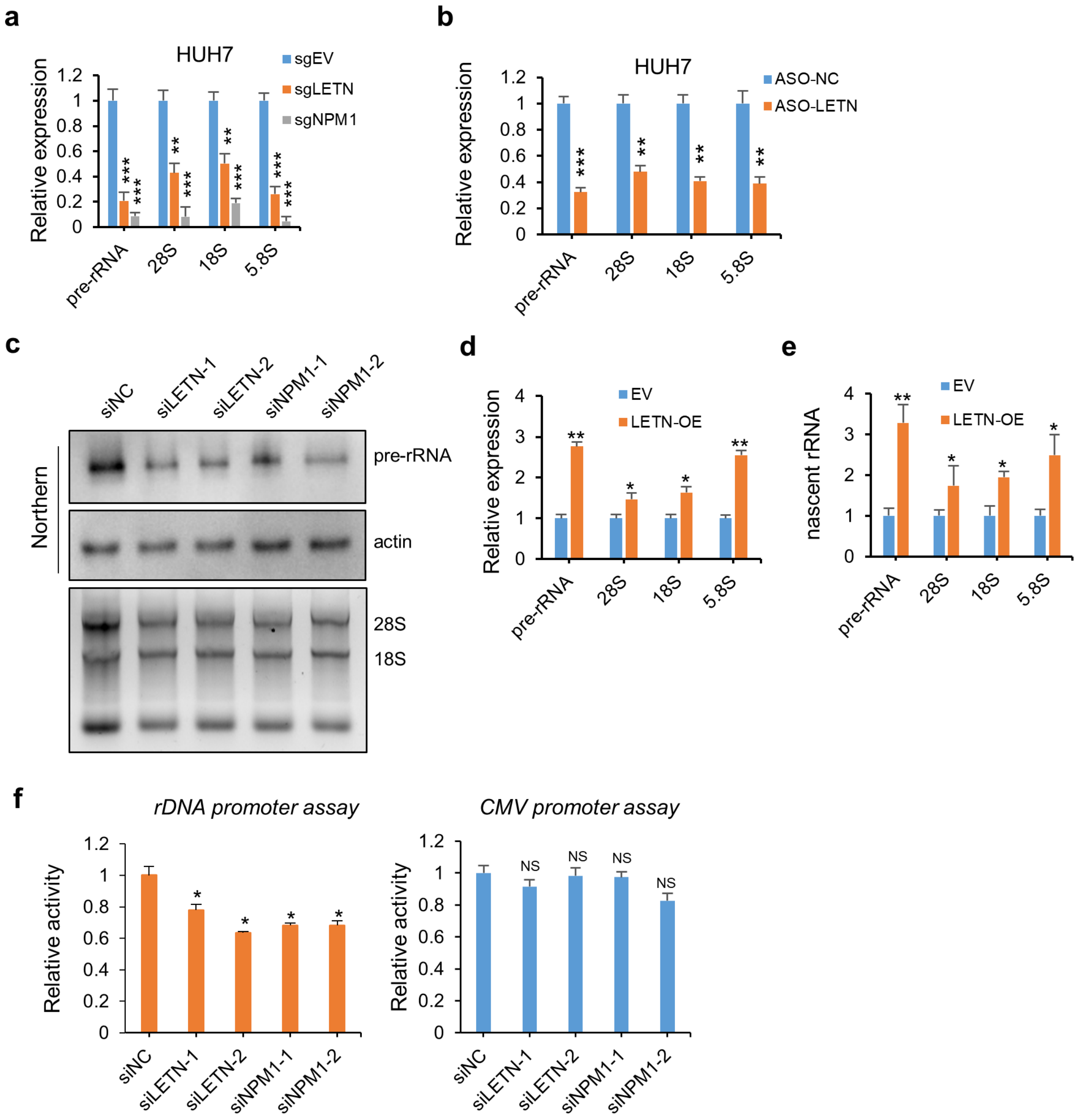

Fig. S17: Effects of LETN and NPM1 on rRNA expression

a Relative expression levels of pre- and mature rRNAs measured by RT-qPCR in HUH7 cells upon CRISPR-mediated LETN or NPM1 knockout. Data shows mean ± SD of 3 biological replicates.

b Relative expression levels of pre- and mature rRNAs measured by RT-qPCR in HUH7 cells upon ASO-mediated LETN knockdown. Data shows mean ± SD of 3 biological replicates.

c Top: Northern blots with the probe for the 47S pre-rRNA from HUH7 cells upon siRNA-mediated LETN or NPM1 knockdown. Total RNA from the same number of cells under different conditions were used as inputs. Beta-actin mRNA was used as a loading control. Bottom: Total RNA resolved by regular gel electrophoresis was also provided to show the RNA integrity.

d Relative expression levels of pre- and mature rRNAs measured by RT-qPCR in HUH7 cells with LETN over-expression. Data shows mean ± SD of 3 biological replicates.

e Relative expression levels of the newly synthesized nascent pre- and mature rRNAs, which were marked by EU and measured by RT-qPCR, in HUH7 cells upon LETN over-expression. Data shows mean ± SD of 3 biological replicates.

f rDNA promoter activity, assessed by relative activity of firefly luciferase, upon LETN or NPM1 knockdown. The CMV promoter activity was measured by Renilla luciferase and used as a reference.
