# Supplementary material for: Mutual dependency between lncRNA LETN and protein NPM1 in controlling the nucleolar structure and functions sustaining cell proliferation
Source: Cell Res. 2021 Jan 11;31(6):664–83. doi: 10.1038/s41422-020-00458-6 (PMC8169757; doi:10.1038/s41422-020-00458-6)
Supplement: Supplementary file 18 — Supplementary information, Figure S18 [file 41422_2020_458_MOESM18_ESM.pdf]

Figure 18

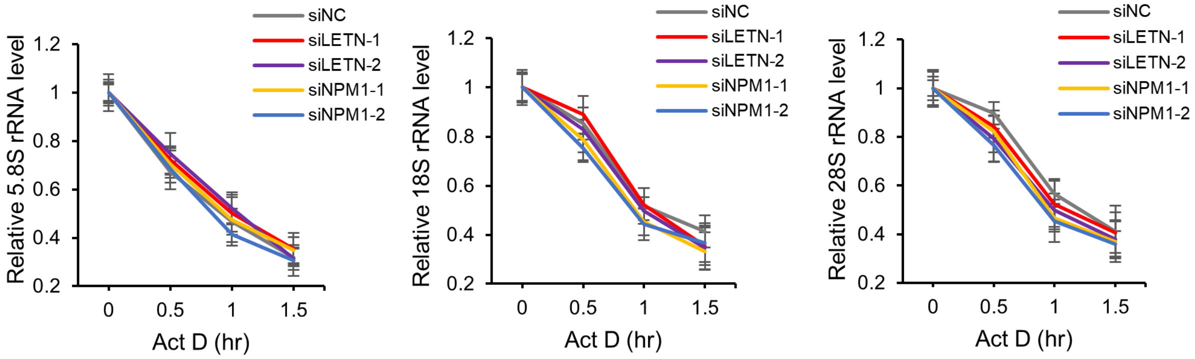

Fig. S18: rRNA degradation dynamics under different conditions.

After treatment of HUH7 cells by actinomycin D (4 uM), which blocked the general transcription, relative expression levels of different rRNAs were measured by qPCR at different time points. The error bars represent the ± SD of 3 biological replicates.
