# Supplementary material for: Mutual dependency between lncRNA LETN and protein NPM1 in controlling the nucleolar structure and functions sustaining cell proliferation
Source: Cell Res. 2021 Jan 11;31(6):664–83. doi: 10.1038/s41422-020-00458-6 (PMC8169757; doi:10.1038/s41422-020-00458-6)
Supplement: Supplementary file 19 — Supplementary information, Figure S19 [file 41422_2020_458_MOESM19_ESM.pdf]

Figure S19

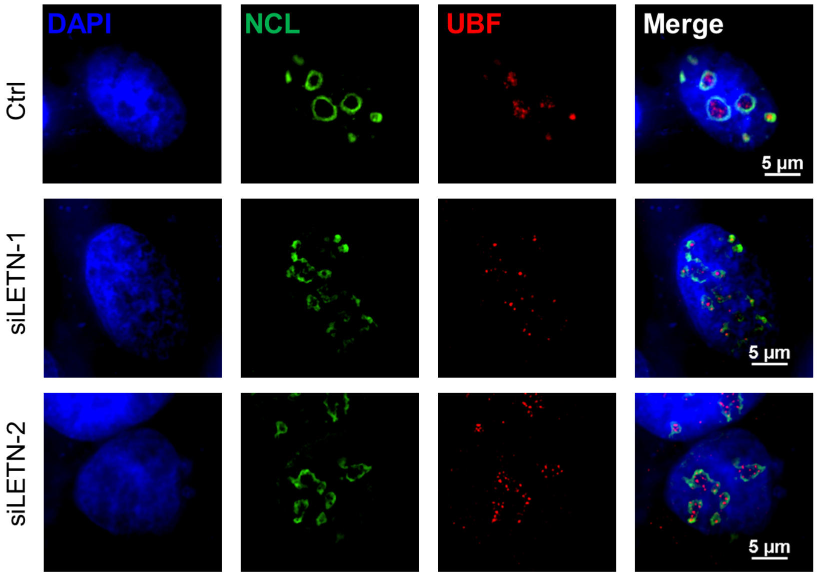

Fig. S19: SIM imges of the nucleolar structures marked by NCL and UBF.

SIM images showing the nucleus staining by DAPI (blue) and IF of NCL (green) and UBF (red) in HUH7 cells.
