# Supplementary material for: Mutual dependency between lncRNA LETN and protein NPM1 in controlling the nucleolar structure and functions sustaining cell proliferation
Source: Cell Res. 2021 Jan 11;31(6):664–83. doi: 10.1038/s41422-020-00458-6 (PMC8169757; doi:10.1038/s41422-020-00458-6)
Supplement: Supplementary file 20 — Supplementary information, Figure S20 [file 41422_2020_458_MOESM20_ESM.pdf]

Figure 20

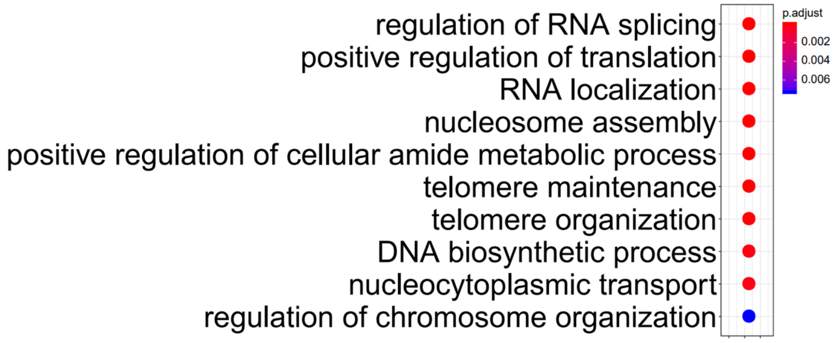

Fig. S20: Functional enrichment of the NPM1-binding proteins.

Enrichments of GO and KEGG functional annotations in the NPM1-binding proteins obtained by MS after NPM1-IP in HUH7 cells.
