# Supplementary material for: Mutual dependency between lncRNA LETN and protein NPM1 in controlling the nucleolar structure and functions sustaining cell proliferation
Source: Cell Res. 2021 Jan 11;31(6):664–83. doi: 10.1038/s41422-020-00458-6 (PMC8169757; doi:10.1038/s41422-020-00458-6)
Supplement: Supplementary file 21 — Supplementary information, Figure S21 [file 41422_2020_458_MOESM21_ESM.pdf]

Figure S21

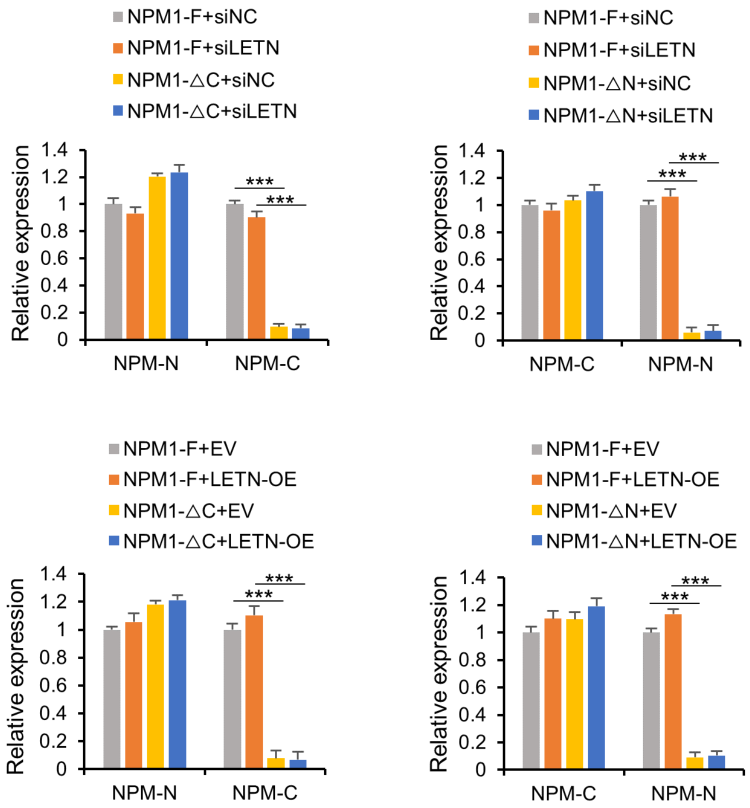

Fig. S21: Rescue of NPM1 by full length or truncated NPM1 in HUH7 cells.

Full length or truncated NPM1 was reintroduced into the $NPM1^{-/-}$ HUH7 cells. qPCR assays were performed with primers that are specific to the N- or C-terminus of NPM1. Data show mean ± SD of 3 biological replicates.
