# Supplementary material for: Mutual dependency between lncRNA LETN and protein NPM1 in controlling the nucleolar structure and functions sustaining cell proliferation
Source: Cell Res. 2021 Jan 11;31(6):664–83. doi: 10.1038/s41422-020-00458-6 (PMC8169757; doi:10.1038/s41422-020-00458-6)
Supplement: Supplementary file 22 — Supplementary information, Figure S22 [file 41422_2020_458_MOESM22_ESM.pdf]

Figure S22

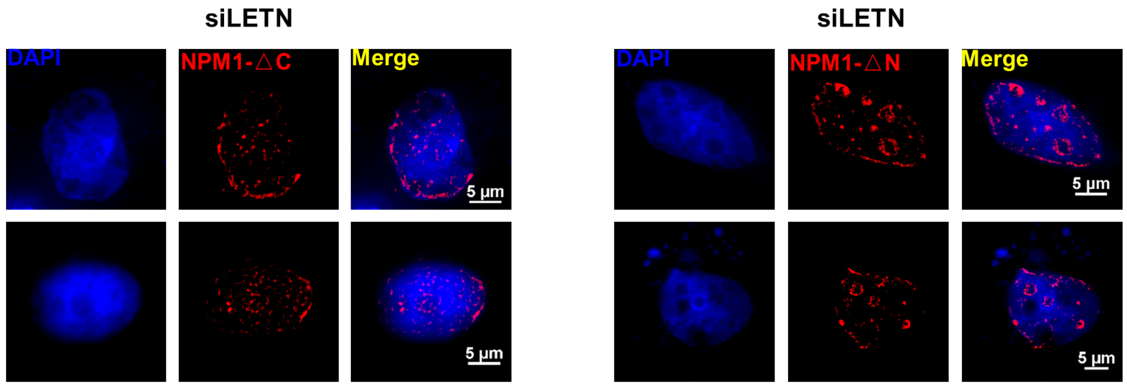

Fig. S22: Distribution patterns of the truncated NPM1 in HUH7 cells upon LETN knockdown.

SIM images showing the nucleus staining by DAPI (blue) and mCherry-labeled NPM1-ΔC or NPM1-ΔN in the NPM1$^{-/-}$ HUH7 cells under the condition of LETN knockdown.
