# Supplementary material for: Mutual dependency between lncRNA LETN and protein NPM1 in controlling the nucleolar structure and functions sustaining cell proliferation
Source: Cell Res. 2021 Jan 11;31(6):664–83. doi: 10.1038/s41422-020-00458-6 (PMC8169757; doi:10.1038/s41422-020-00458-6)
Supplement: Supplementary file 23 — Supplementary information, Figure S23 [file 41422_2020_458_MOESM23_ESM.pdf]

Figure S23

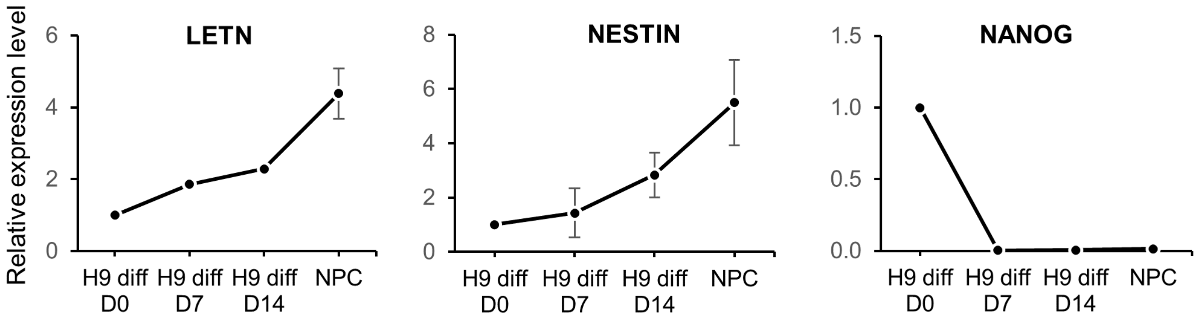

Fig. S23: Relative expressions of LETN and other markers during differentiation from human ESC cells to the NPC cells.

Relative expression levels of LETN measured with qPCR, at different stages during differentiation from the human ESC H9 cells to the NPC cells. NESTIN serves as an NPC marker and NANOG as an ESC marker. Data show mean ± SD of 3 biological replicates.
