# Supplementary material for: Mutual dependency between lncRNA LETN and protein NPM1 in controlling the nucleolar structure and functions sustaining cell proliferation
Source: Cell Res. 2021 Jan 11;31(6):664–83. doi: 10.1038/s41422-020-00458-6 (PMC8169757; doi:10.1038/s41422-020-00458-6)
Supplement: Supplementary file 24 — Supplementary information, Figure S24 [file 41422_2020_458_MOESM24_ESM.pdf]

Figure S24

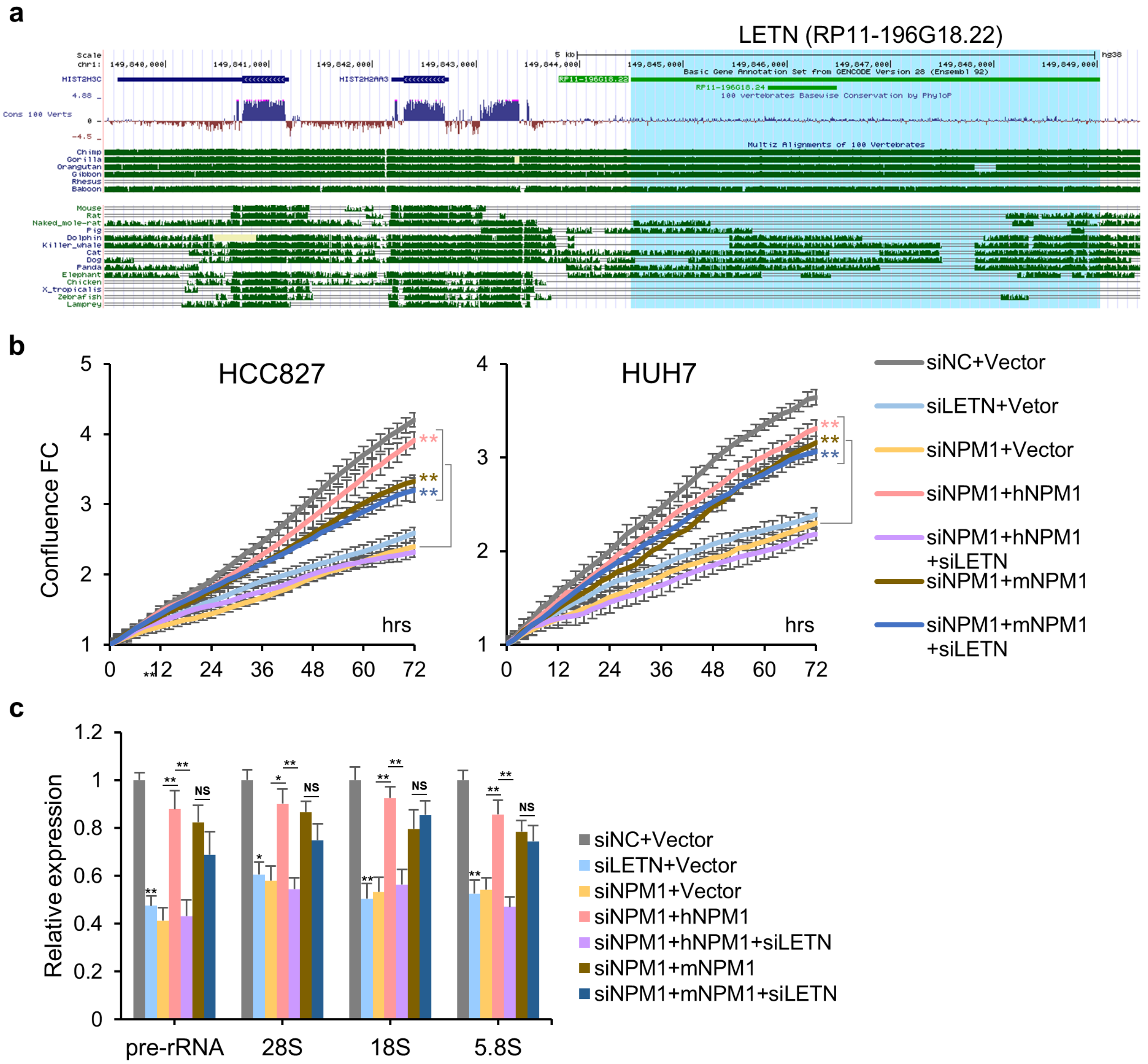

Fig. S24: Cross-species conservation of LETN and comparison between mouse and human NPM1 in terms of their dependency on LETN.

a Genomic location of LETN on the GRCh38/hg38 reference and the cross-species conservation. *Cons 100 Verts*: Base-wide conservation by PhyloP across 100 vertebrates. Multiz alignments of some selected vertebrates are shown, including primates and other representative animals such as mouse, rat, and others. Image was generated with UCSC Genome Browser.

b Proliferation curves of HUH7 cells after NPM1 knockdown and rescue with human or mouse NPM1. The error bars represent the ± SD of 3 biological replicates.

c After knockdown of NPM1 in HUH7 cells, its expression was rescued with human or mouse NPM1. Relative expression levels of pre- and mature rRNAs were measured by RT-qPCR. Data shows mean ± SD of 3 biological replicates.
