# Supplementary material for: Mutual dependency between lncRNA LETN and protein NPM1 in controlling the nucleolar structure and functions sustaining cell proliferation
Source: Cell Res. 2021 Jan 11;31(6):664–83. doi: 10.1038/s41422-020-00458-6 (PMC8169757; doi:10.1038/s41422-020-00458-6)
Supplement: Supplementary file 25 — Supplementary information, Figure S25 [file 41422_2020_458_MOESM25_ESM.pdf]

## Figure S25

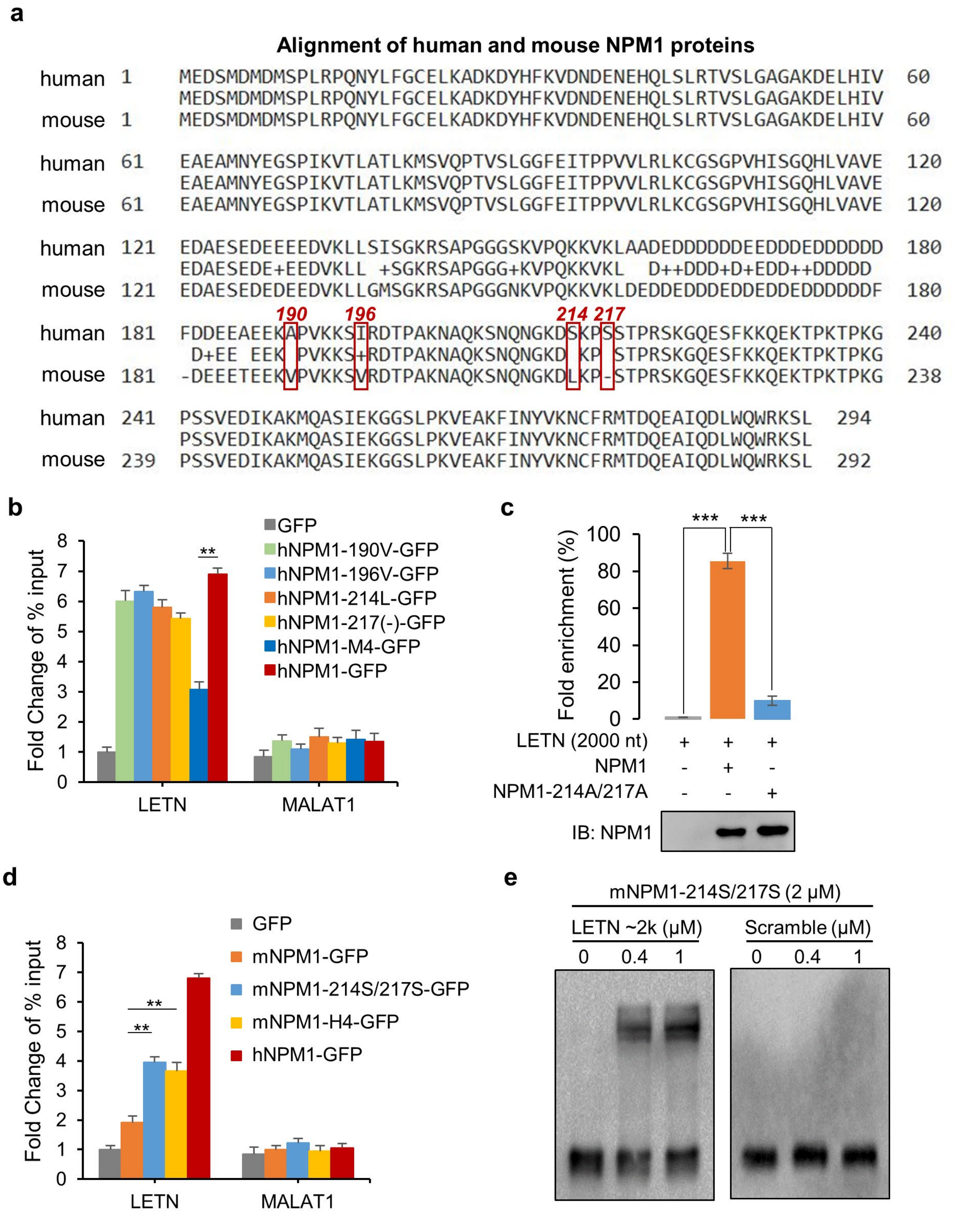


**Fig. S25: Comparison between human and mouse NPM1.**

**a** Alignment of the human and mouse NPM1 proteins, generated by the NCBI protein blast tool.

**b** GFP-tagged wild-type or mutant human NPM1 (hNPM1) at the 4 residues individually or

altogether (M4, i.e., mutation of the 4 residues to the mouse homologue) was reintroduced into NPM1-/- HUH7 cells. qPCR of LETN and MALAT1 was performed after native cross-linking GFP-RIP with the cells. MALAT1 was used as a negative control. The error bars represent the ± SD of 3 biological replicates.

**c** *In vitro* RNA-protein binding assay. LETN fragment from *In vitro* transcription was added to purified NPM1 protein. LETN qRT-PCR was then performed with the material pulled down by anti-NPM1.

**d** GFP-tagged wild-type human NPM1, mouse NPM1, or mutant mouse NPM1 at the 2 residues (214 and 217) or at the 4 residues altogether (H4, i.e., mutation of the 4 residues to the human homologue) was reintroduced into NPM1-/- HUH7 cells. qPCR of LETN and MALAT1 was performed after native cross-linking GFP-RIP with the cells. MALAT1 was used as a negative control. The error bars represent the ± SD of 3 biological replicates.

**e** *In vitro* pentamerization of purified mouse NPM1-214S/217S mutant protein, which was incubated for about 40 minutes with LETN fragment of the first ~2000 nt or scrambled RNA with a similar length. Images of native PAGE with anti-NPM1 show the oligomeric and monomeric NPM1 proteins.
