# Supplementary material for: Mutual dependency between lncRNA LETN and protein NPM1 in controlling the nucleolar structure and functions sustaining cell proliferation
Source: Cell Res. 2021 Jan 11;31(6):664–83. doi: 10.1038/s41422-020-00458-6 (PMC8169757; doi:10.1038/s41422-020-00458-6)
Supplement: Supplementary file 26 — Supplementary information, Figure S26 [file 41422_2020_458_MOESM26_ESM.pdf]

Figure S26

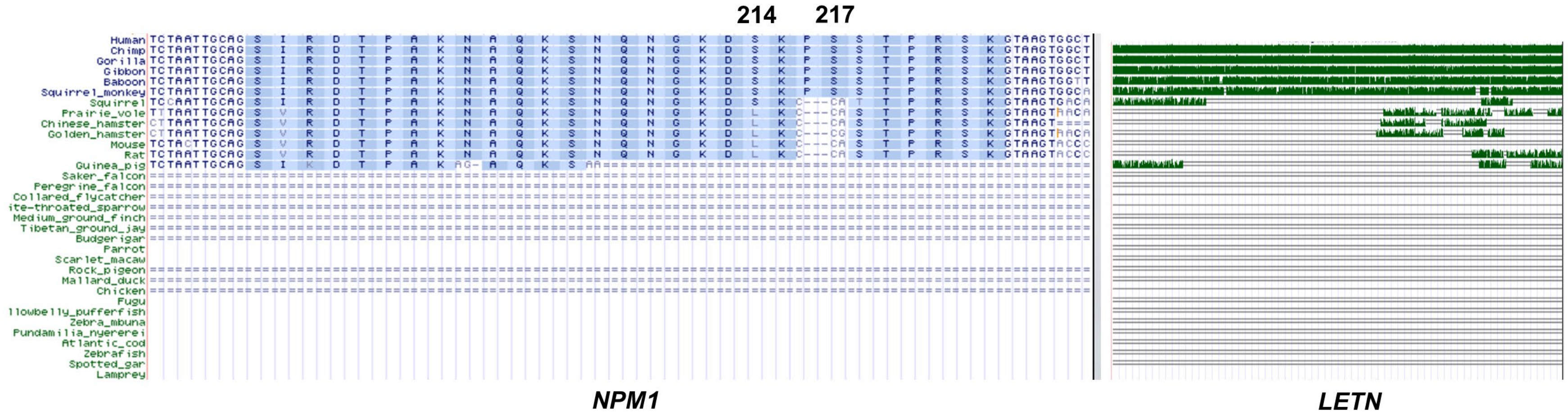

Fig. S26: Conservation of NPM1 and LETN sequences across species.

Alignment of the NPM1 amino acid sequences around the residues 214 and 217 and Multiz alignment of LETN across some representative species.
