# Supplementary material for: Mutual dependency between lncRNA LETN and protein NPM1 in controlling the nucleolar structure and functions sustaining cell proliferation
Source: Cell Res. 2021 Jan 11;31(6):664–83. doi: 10.1038/s41422-020-00458-6 (PMC8169757; doi:10.1038/s41422-020-00458-6)
Supplement: Supplementary file 36 — Supplementary Video Legend [file 41422_2020_458_MOESM36_ESM.pdf]

Supplementary information, Video legend

Video S1-5: Time-lapse immunofluorescence microscopy of the HUH7 cells.

Time-lapse microscope images of HUH7 cells in 5 different fields, showing the mCherry-labeled NPM1 (red) and MS2-tagged LETN marked by MS2-GFP fusion protein (green). The images were captured per 15 minutes for up to 12.5 hours. Figs. 2e and S13b showed some selected snapshots at different time points in Video S1.

Video S6-9: 3-D nucleolar structures reconstructed from SIM images.

3-D reconstruction of the SIM images from immunofluorescence staining of the endogenous NPM1 (green) and Fibrillarin (red) in Videos S6 and S8, or NCL (green) and UBF (red) in Videos S7 and S9, in the HUH7 cells under the normal condition (Videos S6 and S7) or LETN knockdown (Videos S8 and S9). The frame size of the images is 25 µm x 25 µm, and the heights of the reconstructed cuboids are 4.6 µm (Video S6), 5.6 µm (Video S7), 6.4 µm (Video S8), and 5.2 µm (Video S9).
